# The role and function of IκKα/β in monocyte impairment

**DOI:** 10.1038/s41598-020-68018-x

**Published:** 2020-07-22

**Authors:** Norman J. Galbraith, Sarah A. Gardner, Samuel P. Walker, Patrick Trainor, Jane V. Carter, Campbell Bishop, Harshini Sarojini, Stephen J. O’Brien, Aruni Bhatnagar, Hiram C. Polk, Susan Galandiuk

**Affiliations:** 1 Price Institute of Surgical Research, Department of Surgery, University of Louisville School of Medicine, Louisville, KY 40292 USA; 2 Diabetes and Obesity Center, Institute of Molecular Cardiology, University of Louisville School of Medicine, Louisville, KY USA

**Keywords:** Cell signalling, Monocytes and macrophages, Trauma, Surgery, Innate immunity

## Abstract

Following major trauma, sepsis or surgery, some patients exhibit an impaired monocyte inflammatory response that is characterized by a decreased response to a subsequent bacterial challenge. To investigate this poorly understood phenomenon, we adopted an in-vitro model of endotoxin tolerance utilising primary human CD14 + monocytes to focus on the effect of impairment on IκKα/β, a critical part of the NFκB pathway. Impaired monocytes had decreased IκKα mRNA and protein expression and decreased phosphorylation of the IκKα/β complex. The impaired monocyte secretome demonstrated a distinct cytokine/chemokine footprint from the naïve monocyte, and that TNF-α was the most sensitive cytokine or chemokine in this setting of impairment. Inhibition of IκKα/β with a novel selective inhibitor reproduced the impaired monocyte phenotype with decreased production of TNF-α, IL-6, IL-12p70, IL-10, GM-CSF, VEGF, MIP-1β, TNF-β, IFN-α2 and IL-7 in response to an LPS challenge. Surgical patients with infection also exhibited an impaired monocyte phenotype and had decreased SITPEC, TAK1 and MEKK gene expression, which are important for IκKα/β activation. Our results emphasize that impaired monocyte function is, at least in part, related to dysregulated IκKα/β activation, and that IκKα/β is likely involved in mounting a sufficient monocyte inflammatory response. Future studies may wish to focus on adjuvant therapies that augment IκKα/β function to restore monocyte function in this clinically important problem.

## Introduction

Trauma, sepsis and major surgery lead to the release of danger-associated and pathogen-associated molecular patterns^1^. In some patients, the stimulation of toll-like receptors (TLR) may contribute to an excessive inflammatory response and multiple organ failure^2,3^. Such a major physiological insult can also lead to impaired or defective innate immune responses^5^. The improvements in the management of critically ill patients has increased the number of patients surviving the early pro-inflammatory phase, however many patients still die with associated immunosuppression. This form of immune dysfunction has a variable time of onset and impairs the host’s ability to mount a secondary inflammatory response, resulting in failed pathogen clearance and often with overwhelming infection^2,6^. On a cellular level, impaired monocyte function has been highly predictive of secondary infection and mortality following major trauma. This defect overlaps the frequently described endotoxin tolerance, characterized by decreased TNF-α production in response to lipopolysaccharide (LPS) or decreased monocyte HLA-DR expression.

The blunted immune response that occurs as a result of this phenomenon is in part attributed to negative regulation of signalling pathways such as that of Nuclear Factor Kappa-B (NFκB). A study of patients with sepsis has recently demonstrated decreased NFκB activation in response to LPS^7^. Suppression of NFκB activation can occur at various levels of signalling; from changes in toll-like receptor expression, MyD88 and TNF-associated receptor factor (TRAF) adaptor proteins, to dysregulation of the subunits of the canonical and non-canonical NFκB pathway itself.

Circulating antigen-presenting monocytes allow peptides derived from antigens, such as LPS, to be processed in vesicles and externalized through major histocompatibility complexes (MHC) class II glycoproteins. Human leukocyte antigen (HLA)-DR is the most prominent of the MHC II proteins, which binds to T-cell receptors on T-cells^8^. The expression and ability to display cell surface HLA-DR is downregulated during a “reprogramming” in response to pathological immune environments, and this immunoparalysis reflects a decreased ability to engage immune effector cells to induce functional immune responses^9^. Therefore, decreased monocyte HLA-DR has been accepted as a reliable and reproducible marker in patients with sepsis, trauma, burns and peri-operative immune status, correlating with mortality^10^.

A sufficient pro-inflammatory cytokine response relies, at least in part, on the propagation of a TLR ligand binding to its receptor with phosphorylation of the Inhibitor of Kappa-B Kinase (IκK)-α/β causing a conformational change which then phosphorylates IκBα. Ubiquitination and proteosomal degradation of IκBα may then follow, allowing the translocation of the active NFκB subunits such as RelA to the nucleus. The exact function and role of IκK-α and IκK-β in monocyte impairment are not fully understood.

The IκK complex contains two catalytic serine/threonine-specific subunits, IκK-α and IκK-β, along with the regulatory subunit IκK-γ. The two catalytic subunits are both approximately 750 amino acids in size, with similar structure. Although they perform distinct regulatory roles in canonical and non-canonical pathways, loss-of-function studies indicate that both subunits are required to mount a sufficient pro-inflammatory response^11,12^.

Caecal ligation and puncture (CLP) models have shown that both shock and sepsis lead to an initial phosphorylation of IκKα/β corresponding to the systemic inflammatory response syndrome (SIRS)^13^. Recent studies using a selective inhibitor of IκKα/β to prevent its phosphorylation resulted in decreased cytokine production and decreased end-organ failure^14^. This type of model does not, however, reproduce the well-documented immune suppression that occurs in some patients, and thus the study of the function and role of IκKα/β as a potential target in human monocyte impairment is possibly important.

The aim of the present study was to investigate the expression and functionality of IκK-α & β in primary human monocytes using a model of endotoxin tolerance. Furthermore, we aimed to determine to what degree IκK-α/β inhibition recapitulated the phenotype of impaired monocyte function using a novel selective inhibitor called IκK-16.

## Results

### Low dose LPS exposure leads to a suppressed monocyte cytokine response and decreased HLA-DR expression

**Figure 1:**
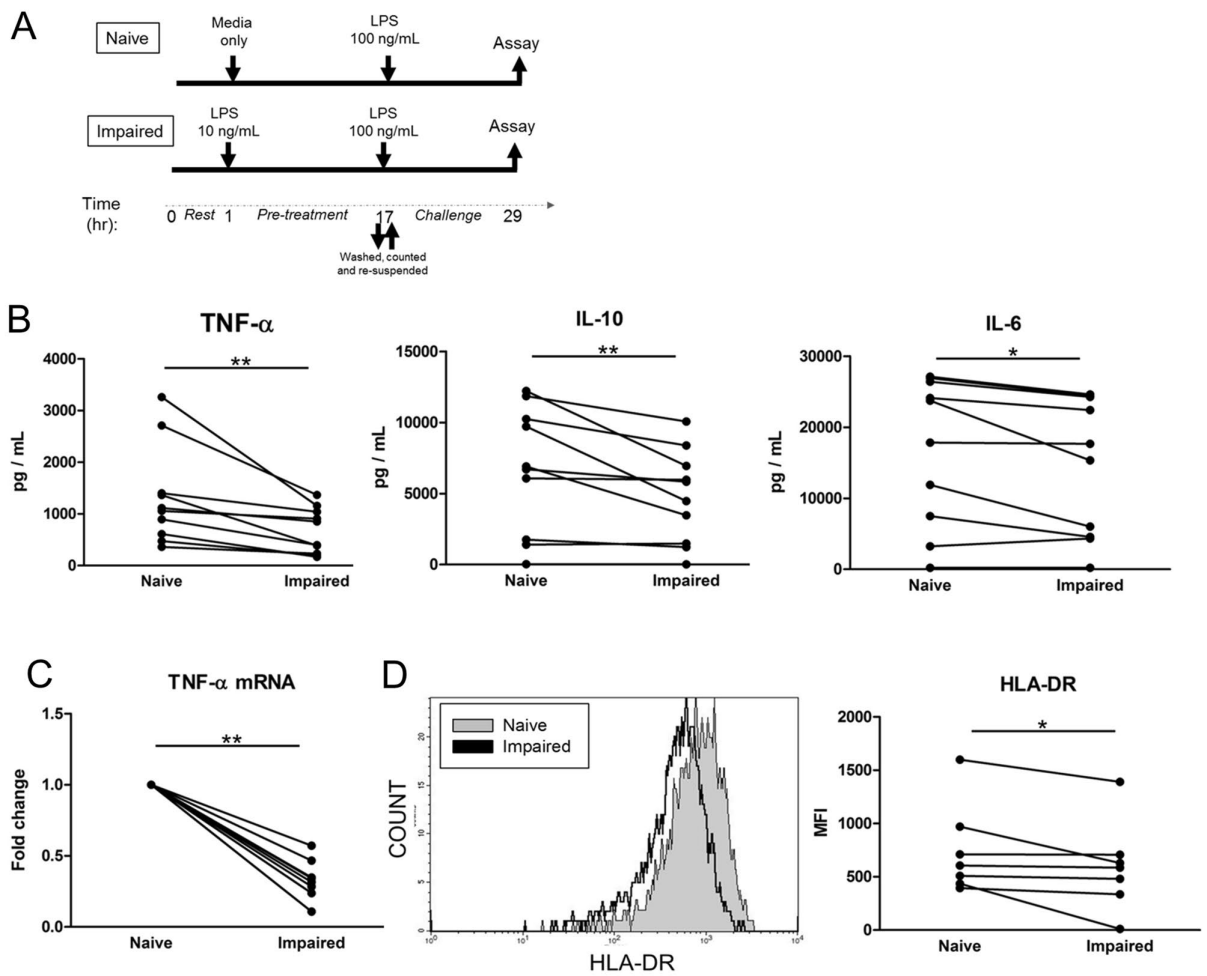
Low dose LPS exposure leads to suppressed cytokine production and decreased HLA-DR expression. Cultured primary human monocytes were treated with 10 ng/mL of LPS (impaired) or media only (naïve) for 16 h, washed and resuspended in fresh media before challenge with 100 ng/mL of LPS. (**A**) Experimental design for endotoxin tolerance model. (**B**) Supernatant concentrations of TNF-α, IL-10 and IL-6 were determined 12 h after the 100 ng/mL LPS challenge (n = 10). (**C**) TNF-α mRNA expression was measured at 2 h after the LPS challenge (n = 8). (**D**) Monocyte surface HLA-DR expression was measured after 16 h of naïve or impaired conditions by flow cytometry. A representative histogram is shown on the left, and quantitative data on the right (n = 7). Data shown as mean ± SE. *p < 0.05, **p < 0.01, Wilcoxon signed-rank test.

In order to study the molecular mechanisms underpinning monocyte impairment, we adopted the approach of isolating CD14 monocytes from whole blood of healthy volunteers and used a model of endotoxin tolerance similar to that of other groups^15,16^. Our experience with this model regarding the purity and time course of the suppressed cytokine response has been previously reported^17,18^. In isolated monocytes exposed to a low dose of LPS (10 ng/mL), there is a suppressed ability to produce TNF-α, IL-10 and IL-6 protein following an LPS challenge (100 ng/mL) (p < 0.05) when compared to the naïve monocyte not exposed to low dose LPS stimulation (Fig. 1A,B). Furthermore, impaired monocytes have decreased TNF-α gene expression as well as lower HLA-DR surface expression (p < 0.05) (Fig. 1C,D).

### TNF-α production was the cytokine most suppressed in the impaired monocyte

**Figure 2:**
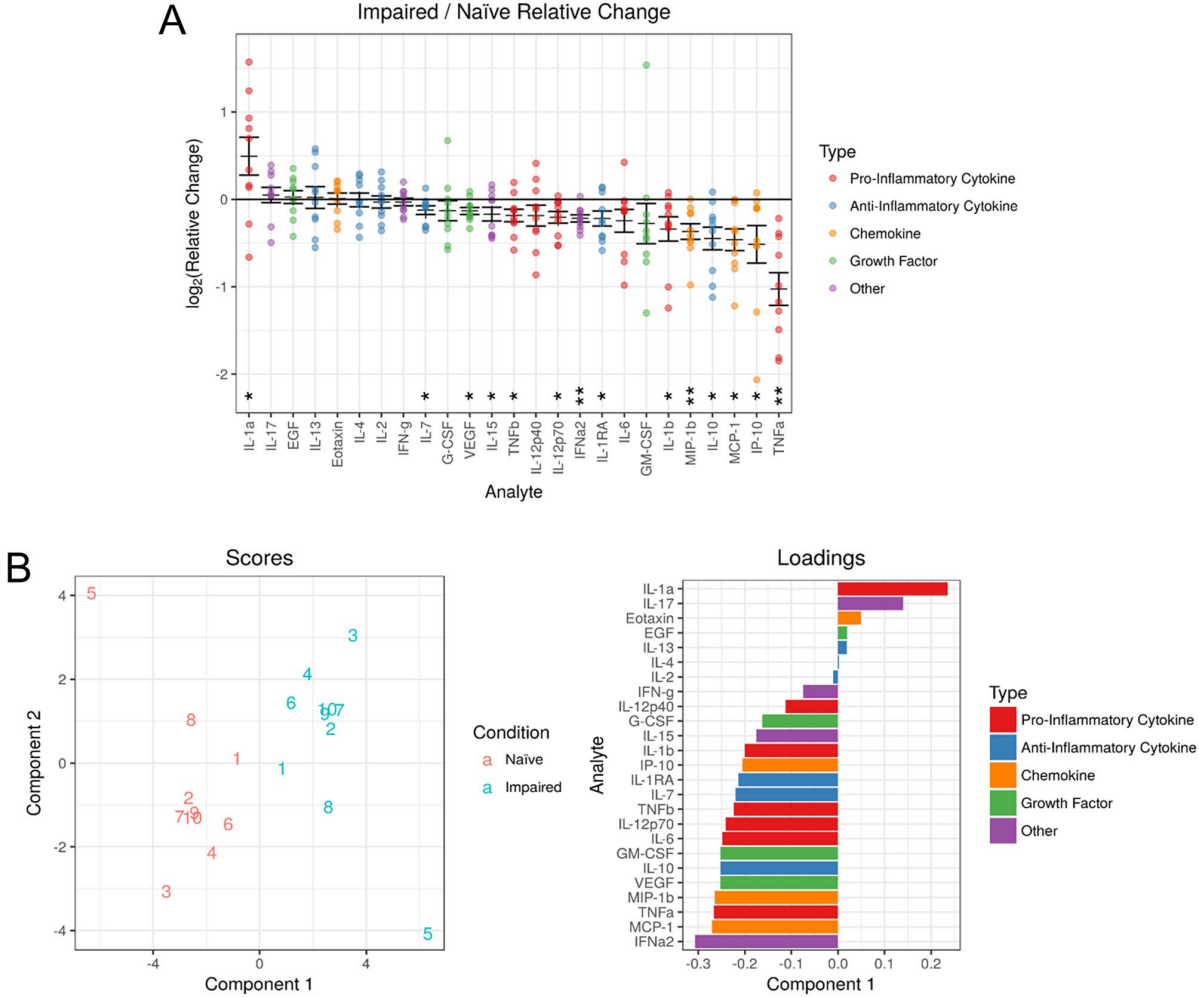
Impaired monocytes have a distinct cytokine/chemokine signature with TNF-α production being the most profoundly suppressed cytokine. Cell culture supernatants were collected 12 h after the 100 ng/mL LPS challenge, comparing 10 ng/mL of LPS (impaired) against media only (naïve) conditions. (**A**) Each cytokine and chemokine is plotted according to its relative change from naïve to impaired conditions, where each dot represents a within-donor relative change. Error bars denote mean ± SE. Statistical significance is denoted by * for q < 0.05 and ** for q < 0.01. (**B**) Multilevel partial least squares discriminant analysis (PLS-DA) comparing the naive (green) and impaired (red) conditions. Left: Scores plot shows the individual sample values latent component scores. Right: Analyte contributions to defining the latent components. Data based on 10 donors.

The impaired monocyte was compared to the naïve control by profiling cytokine and chemokine differences 12 h after stimulation from the 100 ng/mL LPS challenge using the multiplex assay. Analytes that were significantly suppressed in impaired monocyte conditions included pro-inflammatory cytokines (TNF-α, IL-1β, IL-12p70 and TNF-β), anti-inflammatory cytokines (IL-10, IL-1RA and IL-7), chemokines (MCP-1, IP-10 and MIP-1β) and others (VEGF, IFN-α2 and IL-15) (q < 0.05) (Fig. 2A). TNF-α was the most consistently different cytokine with the largest magnitude of suppression. Interestingly, IL-1α production was consistently increased in the impaired monocyte as compared to the naïve control. Cytokine and chemokine levels across conditions are shown in Supplementary Table 1.

### Endotoxin tolerance is a distinct phenomenon based on changes in cytokine and chemokine production

Analysis of cytokine secretion between naïve and impaired monocytes by paired PLS-DA demonstrated distinct secretion patterns (Fig. 2B). The five-fold cross-validation estimated AUC for the estimated paired PLS-DA model using the loadings seen in Fig. 2B was 1.00, predicting the difference between naïve and impaired conditions in all cases.

### Impaired monocytes exhibit diminished IκBα degradation and decreased IκKα/β phosphorylation

**Figure 3:**
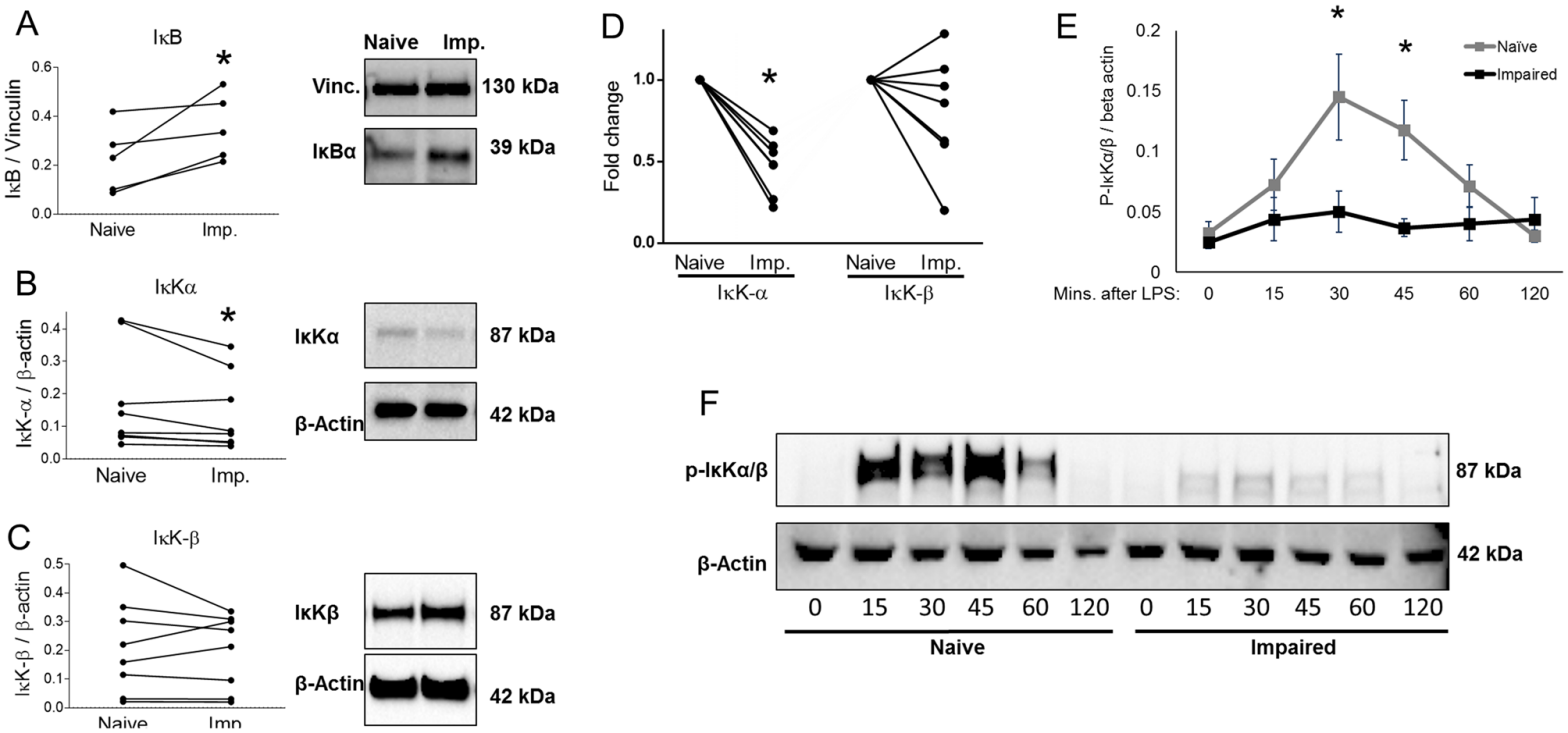
IκKα/β signaling in the impaired monocyte. (**A**) Primary monocytes were cultured in either media only (naïve) or 10 ng/mL of LPS for 16 h, washed and resuspended in fresh media and challenged with 100 ng/mL of LPS for 60 min. Total protein was analyzed by Western blots for IκBα and normalized to vinculin (n = 5). (**B**, **C**) Primary monocytes were cultured for 16 h in media only (naïve) or 10 ng/mL of LPS (impaired) and total protein concentrations of IκK-α & IκK-β were determined by normalizing to β-actin (n = 8 for each). (**D**) After 16 h of incubation in naïve or impaired conditions, IκK-α & IκK-β mRNA expression was measured by qRT-PCR normalizing to 18S (n = 7). (**E**, **F**) Monocytes were cultured in naïve or impaired conditions for 16 h, washed and then challenged with 100 ng/mL of LPS. Phosphorylation of IκKα/β was determined by Western blot and normalized to β-actin (n = 6). Original uncropped gels are available in Supplemental Material. Data is shown as mean ± SE. *p < 0.05, Wilcoxon signed-rank test.

**Figure 4:**
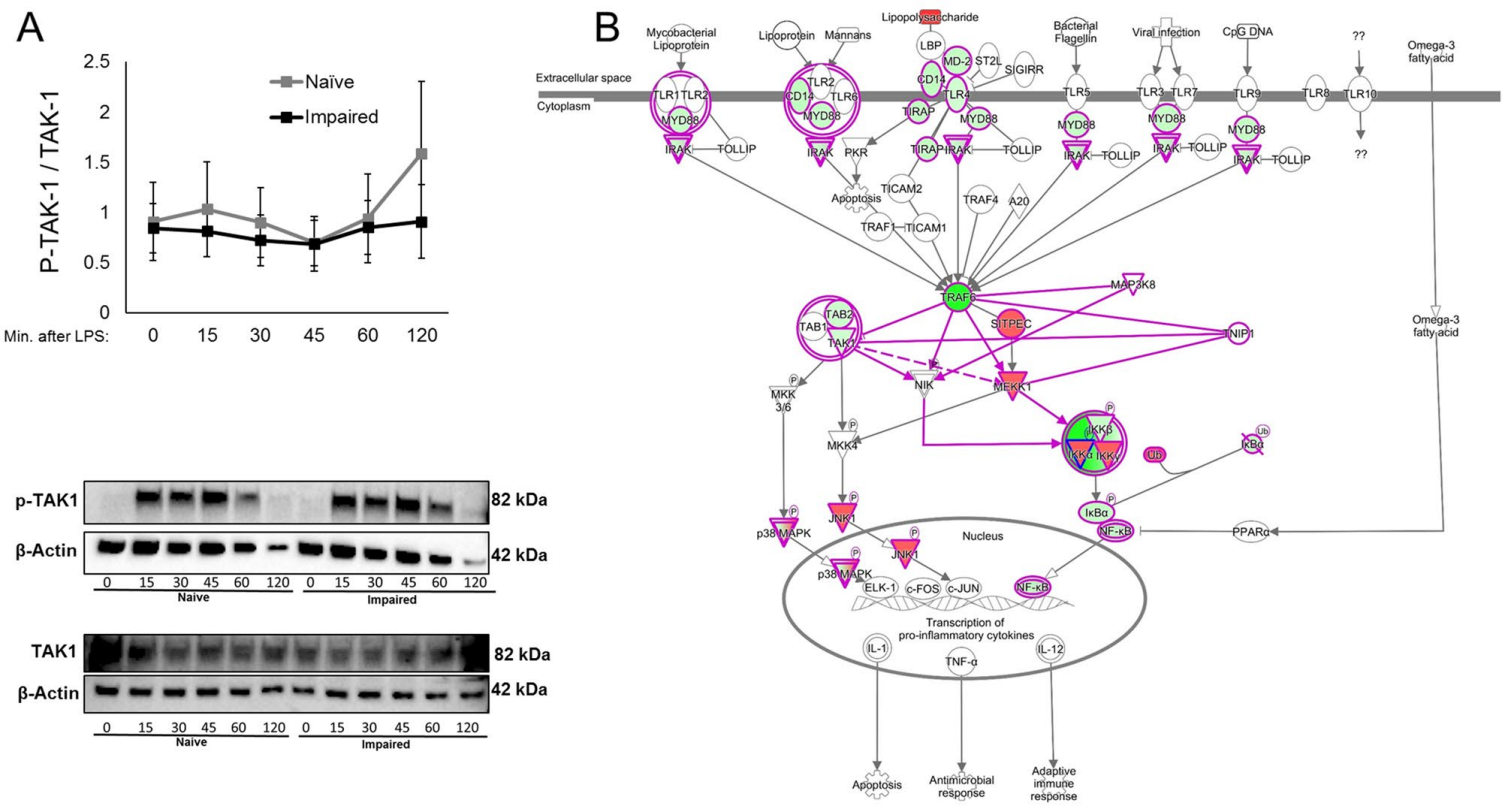
Upstream signalling in the impaired monocyte. (**A**) Monocytes were cultured in naïve or impaired conditions for 16 h, washed and then challenged with 100 ng/mL of LPS. TAK-1 activation was expressed as a ratio of phosphorylated over total TAK-1 levels, normalized to β-actin (n = 5). Original uncropped 15-well gels are available in Supplemental Material. For each individual sample and protein, different β-actin levels are presented because separate Western Blots were performed on the same samples to avoid antibody interactions. Data is shown as mean ± SE. *p < 0.05, Wilcoxon signed-rank test. (**B**) Pathway analysis of toll-like receptor signaling in the impaired monocyte based on gene expression profiling. The pathway analyses were generated through the use of IPA (QIAGEN Inc., https://www.qiagenbioinformatics.com/products/ingenuity-pathway-analysis)4.

The cytokines that were significantly different in the impaired monocyte as compared to the naïve monocyte were entered into a network analysis (Metacore®, https://portal.genego.com) in order to determine affected signalling pathways. The signalling network with the highest prediction score was NFκB (Z-score 323.72). This pathway included 11 seed nodes and is predicted to directly regulate the dysregulated cytokines of the impaired monocyte as well as HLA-DR expression (Supplemental Fig. 2). IκBα degradation in response to stimulation from a 100 ng/mL LPS challenge was diminished in the impaired monocyte (Fig. 3A). Given the evidence of decreased NFκB activation, we interrogated IκKα/β, the upstream kinase, for differences in the level or function of this complex. Both the gene expression and total protein of IκKα was decreased in the impaired monocyte compared with the naïve control (p < 0.05) (Fig. 3B,D). Levels of IκKβ were equivalent between naïve and impaired conditions in terms of gene expression and total protein (Fig. 3C,D). There was an increase in IκKα/β phosphorylation in the naïve monocyte seen maximally at 30 and 45 min following a 100 ng/mL LPS challenge, whereas the same response was not seen in the impaired monocyte (p < 0.05) (Fig. 3E). No differences in either total or phosphorylated forms of TAK-1 were seen between naïve and impaired monocytes (Fig. 4A). Using previously reported toll-like receptor gene expression data of the impaired monocyte^17^, analysis revealed a complex network of signalling change between TRAF-6 and the IKK complex as demonstrated in Fig. 4B.

### IκKα/β inhibition produces a tolerant-like cytokine/chemokine phenotype but does not suppress HLA-DR production

**Figure 5:**
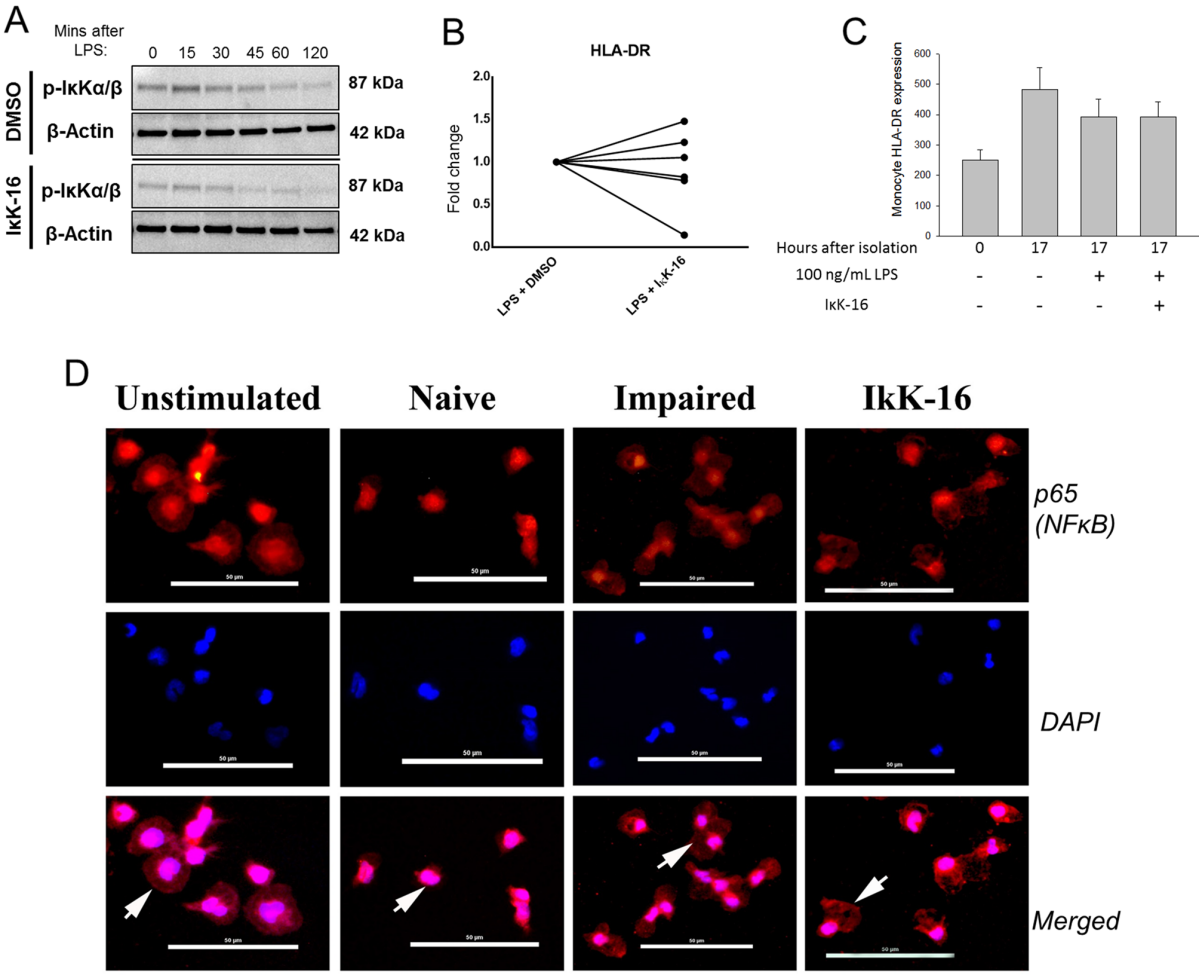
IκK inhibition decreases NFκB activation but not HLA-DR expression. (**A**) An IκK inhibitor, IκK16, decreases the phosphorylation of IκKα/β as determined by Western blot. A representative blot is shown using β-actin as a loading control. Original uncropped 10-well gels are available in Supplemental Material. (**B**) Primary monocytes were cultured for 16 h in conditions of LPS 100 ng/mL and DMSO, or LPS 100 ng/mL and 100 nM IκK-16. HLA-DR mRNA was determined using qRT-PCR with 18S as an internal control. (**C**) Primary monocytes were cultured for 16 h in conditions with or without 100 ng/mL of LPS and 100 nM IκK-16 as indicated. Surface HLA-DR expression was measured by FACS (n = 6). (**D**) Monocytes were cultured for 16 h, washed, resuspended and then cultured for a further 45 min in the following conditions; Unstimulated (media/media), Naïve (media/100 ng/mL LPS), Impaired (10 ng/mL LPS/100 ng/mL LPS), IκK-16 (100 nM IκK-16/100 ng/mL LPS). Representative photomicrographs for p65 (NFκB) co-localisation in each condition are demonstrated. Red: p65 (Texas Red), Blue: nucleus (DAPI). Images are at 40X. Scale bar is 50 μM. White arrows illustrate cytoplasmic or nuclear location of p65 of each condition.

**Figure 6:**
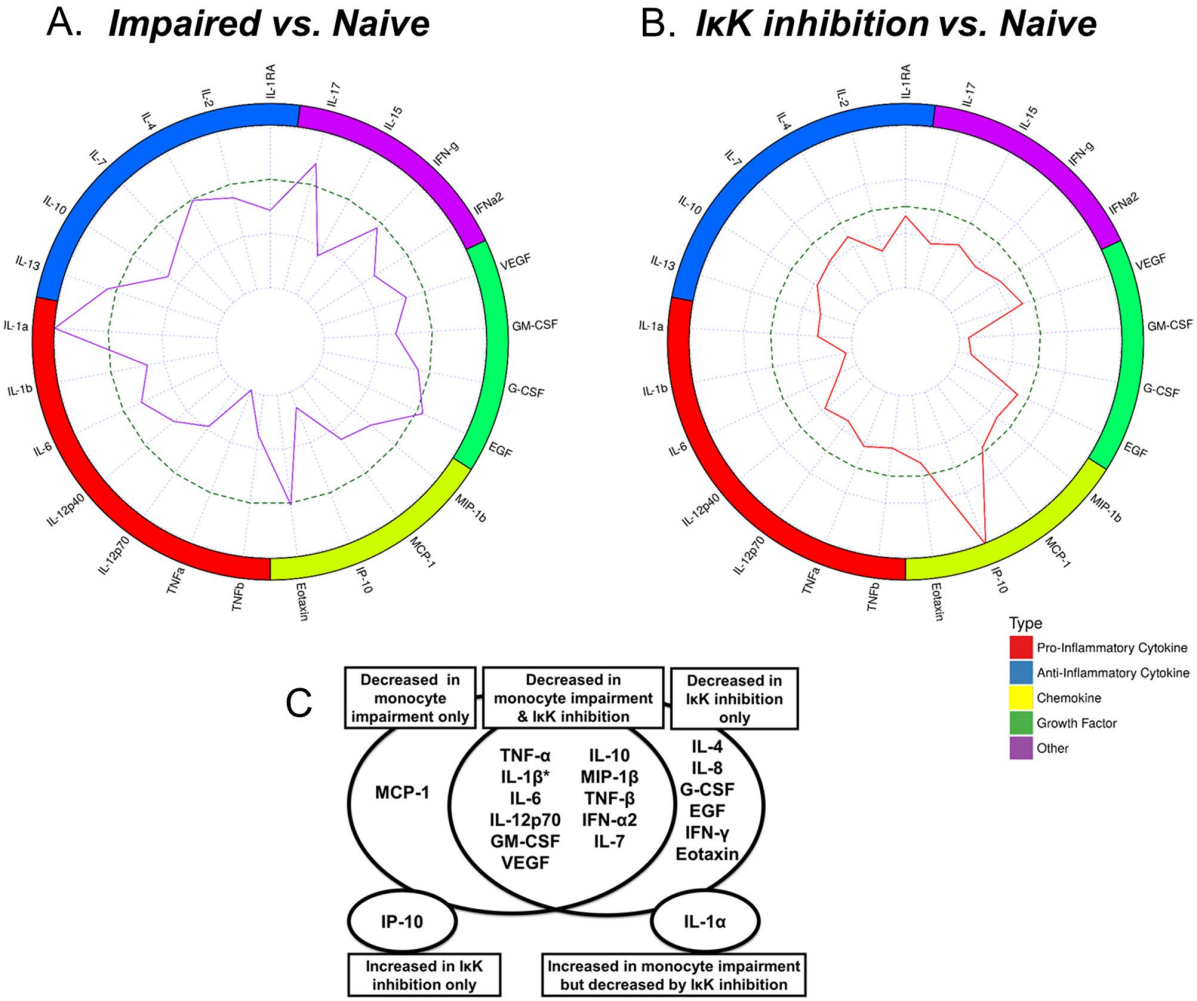
Monocyte impairment has an overlapping cytokine/chemokine footprint with IκKα/β inhibition. Monocytes were cultured for 16 h, washed, resuspended and then cultured for a further 12 h in the following conditions; Unstimulated (media/media), Naïve (media/100 ng/mL LPS), Impaired (10 ng/mL LPS/100 ng/mL LPS), IκK-16 (100 nM IκK-16/100 ng/mL LPS) before cell culture supernatants were collected. Supernatants were analyzed for cytokine and chemokine concentrations by Multiplex cytokine ELISA (Supplemental Table 2) and the relative change compared to unstimulated conditions. Radar plots compare the effect of monocyte impairment (**A**) and the effect of IκK inhibition (**B**) on secretion profiles. Mediators are categorized as pro-inflammatory (red) and anti-inflammatory (blue) cytokines, chemokines (yellow), growth factors (green) and other (purple). Mean log2 (expression) is plotted relative to the naïve monocyte (dotted circle) as its control, based on 10 donors. (**C**) Venn diagram demonstrating the overlapping cytokine/chemokine changes in monocyte impairment (red) and IκKα/β inhibition. IP-10 (bottom left) represents the only mediator increased during IκKα/β inhibition, and IL-1α (bottom right) represents the only mediator increased during monocyte impairment. *p = 0.08 between naïve and impaired conditions.

Given the marked differences in IκKα/β in phosphorylation between naïve and impaired monocytes, we further studied the role of IκKα/β in the monocyte inflammatory response by using IκK-16, a novel selective inhibitor of IκKα/β. This has previously been shown to inhibit the phosphorylation of IκKα/β in animal models of shock and sepsis^13,14^. Incubation of primary monocytes with IκK-16 led to decreased IκK/α/β phosphorylation in response to 100 ng/mL LPS challenge (Fig. 5A). IκK-16 is not toxic to cultured monocytes, even at high doses^17^. Immunofluorescence imaging demonstrated that in naïve monocytes, p65 translocates to the nucleus in response to an LPS challenge (Fig. 5D). In impaired cells, however, p65 is still present in the cytoplasm. Cells treated with IκK-16 similarly have p65 remaining in the cytoplasm, indicating that IκK-16 treatment prevents NFκB activation. We have previously shown that IκK-16 decreases TNF-α and IL-10 production in a dose-related fashion, and that a concentration of 100 nM could sufficiently suppress production of both cytokines^17^. Despite these inhibitory effects on cytokine production, there were no effects of IκK inhibition on HLA-DR gene expression or protein levels (Fig. 5B,C). Inhibition of IκK did not affect levels of IRAK-M expression (Supplementary Fig. 3). Impaired monocytes and IκK-16-treated monocytes were then both compared to the naïve monocyte in terms of their cytokine and chemokine profiles to determine to what degree suppression of IκKα/β phosphorylation contributes to the impaired phenotype (Fig. 6A). Analytes that were commonly suppressed in both monocyte impairment and after IκK inhibition include TNF-α, IL-6, IL-12p70, IL-10, GM-CSF, VEGF, MIP-1β, TNF-β, IFN-α2 and IL-7 (Fig. 6B). Similarly, IL-1β levels approached a significant decrease in impaired and IκK inhibited conditions (p = 0.08 and p < 0.05, respectively). MCP-1 was the only mediator that was suppressed in monocyte impairment, but not with IκK-16 treatment. In addition to the analytes decreased by both IκK inhibition and impaired conditions, further analytes decreased in conditions of IκK inhibition only included IL-4, IL-8, G-CSF, EGF, IFN-γ and eotaxin production. The levels of the latter analytes were similar between naïve and impaired conditions. IP-10 was the only mediator that was increased following IκK inhibition. IFN-β levels were low across all conditions. No differences were seen between naïve and impaired cells but appeared higher following IκK inhibition (p = 0.051).

### Patients with infection have impaired monocyte function and dysregulated IκKα expression

**Figure 7:**
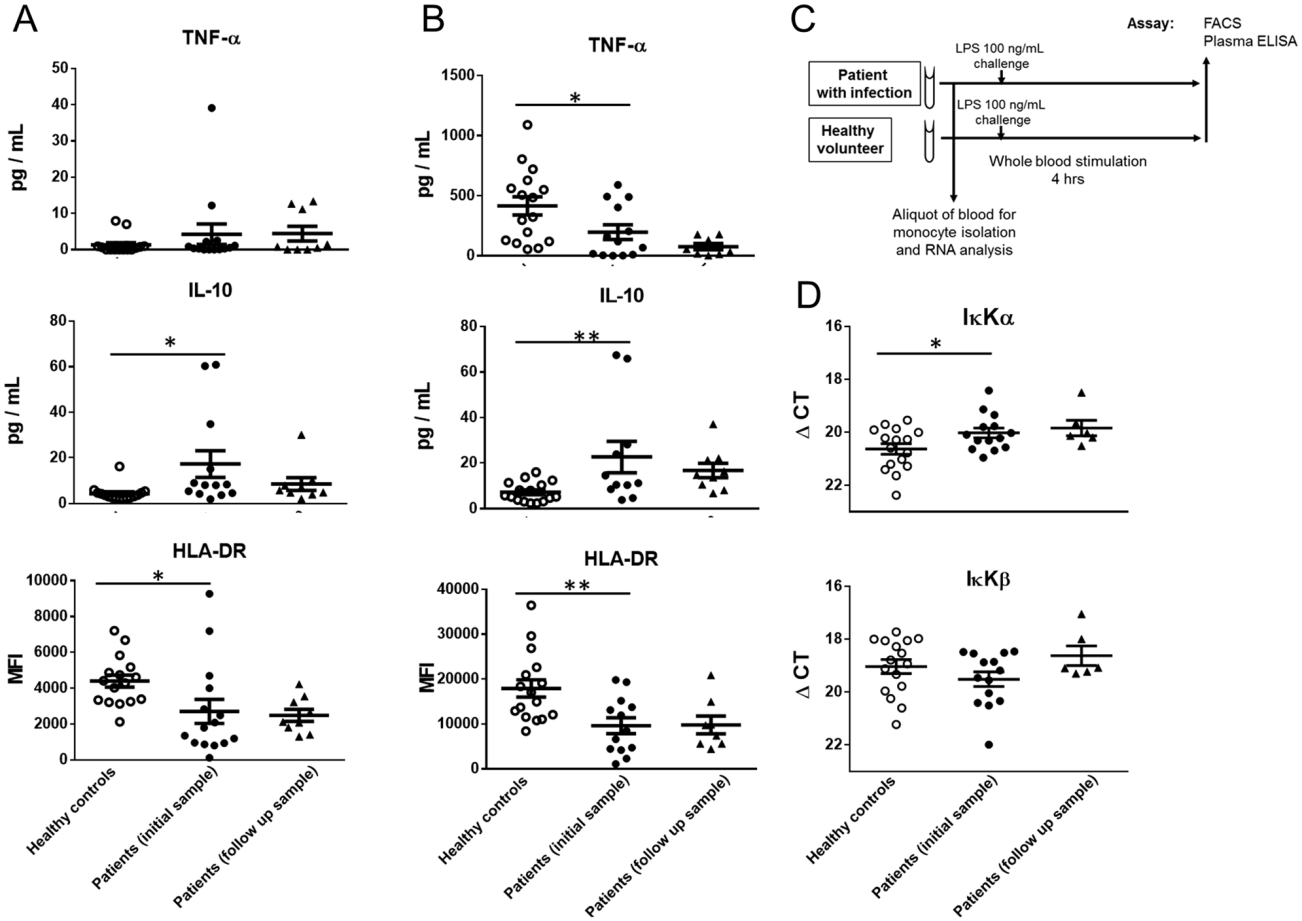
Patients with infection have decreased HLA-DR expression and TNF-α production. Peripheral blood was sampled from patients during infection and after recovery, and compared to that of healthy volunteers. (**A**) Baseline unstimulated levels of TNF-α and IL-10 were determined by ELISA, with monocyte surface HLA-DR measured by flow cytometry. (**B**) Whole blood was stimulated with 100 ng/mL of LPS for 4 h and TNF-α, IL-10 and HLA-DR were measured in the same manner. (**C**) Experimental design for whole blood stimulation assays. (**D**) CD14 monocytes were isolated from whole blood and IκKα and IκKβ mRNA expression was measured with normalization to 18S. **p < 0.01, *p < 0.05, ANOVA with post-hoc Holm–Sidak test.

Surgical patients with infection were studied to determine their same monocyte function. Peripheral whole blood was stimulated with LPS ex-vivo (Fig. 7C) to determine their immune responsiveness, similar to other groups^19,20^. Patients with concurrent infection had a decreased capacity for TNF-α production in response to a 100 ng/mL LPS challenge (p < 0.05) as seen in Fig. 7B, similar to the in-vitro model of impairment. In addition, they had lower baseline monocyte HLA-DR (p < 0.05) and in response to 100 ng/mL LPS stimulation (p < 0.01) (Fig. 7A,B). Increased circulating plasma IL-10 levels were demonstrated in patients as compared to healthy controls (p < 0.05), with further increases in response to 100 ng/mL LPS challenge (p < 0.01) (Fig. 7A,B). Levels of IκKα were elevated in patients during infection as compared with healthy volunteers (p < 0.05), whereas IκKβ levels were unchanged (Fig. 7D). It should be noted that studies with lengthy follow-up in patients with infections are uncommon. Following clinical recovery from infection, IL-10 levels returned towards that of healthy volunteers. Clinical details of surgical patients with infection are described in Table 1.

**Table 1:** Demographics and characteristics of patients with infection.

		Patients (n = 16)
Men, n (%)		9 (56)
Median age, y (± SD)		58.0 (± 13.2)
Systolic blood pressure < 90 mmHg, n (%)		1 (6.3)
Heart rate > 90/min, n (%)		3 (18.8)
Leukocyte count, cells/mL (± SD)		13, 349 (± 9, 705)
Temperature > 100.4ºF, n (%)		2 (12.5)
Respiratory rate > 20/min, n (%)		0 (0)
**Source of infection, n (%)**		
Intra-abdominal abscess		10 (62)
Pneumonia		2 (12.5)
Pelvic abscess		2 (12.5)
Deep ischiorectal abscess		1 (6)
Bacteremia (infected infusion port)		1 (6)
**Co-morbidity, n (%)**		
IBD		5 (31.3)
Cancer		4 (25.0)
Diverticulitis		2 (12.5)
Other		4 (25.0)
Median days from operation to 1st sample (min–max)		11 (0–1585)
Median days from 1st sample to follow up sample (min–max)*		60 (34–208)

IBD, inflammatory bowel disease; SD, standard deviation.

*In patients that underwent follow-up sampling.

Patients with infection have decreased levels of toll-like receptor gene expression.

**Figure 8:**
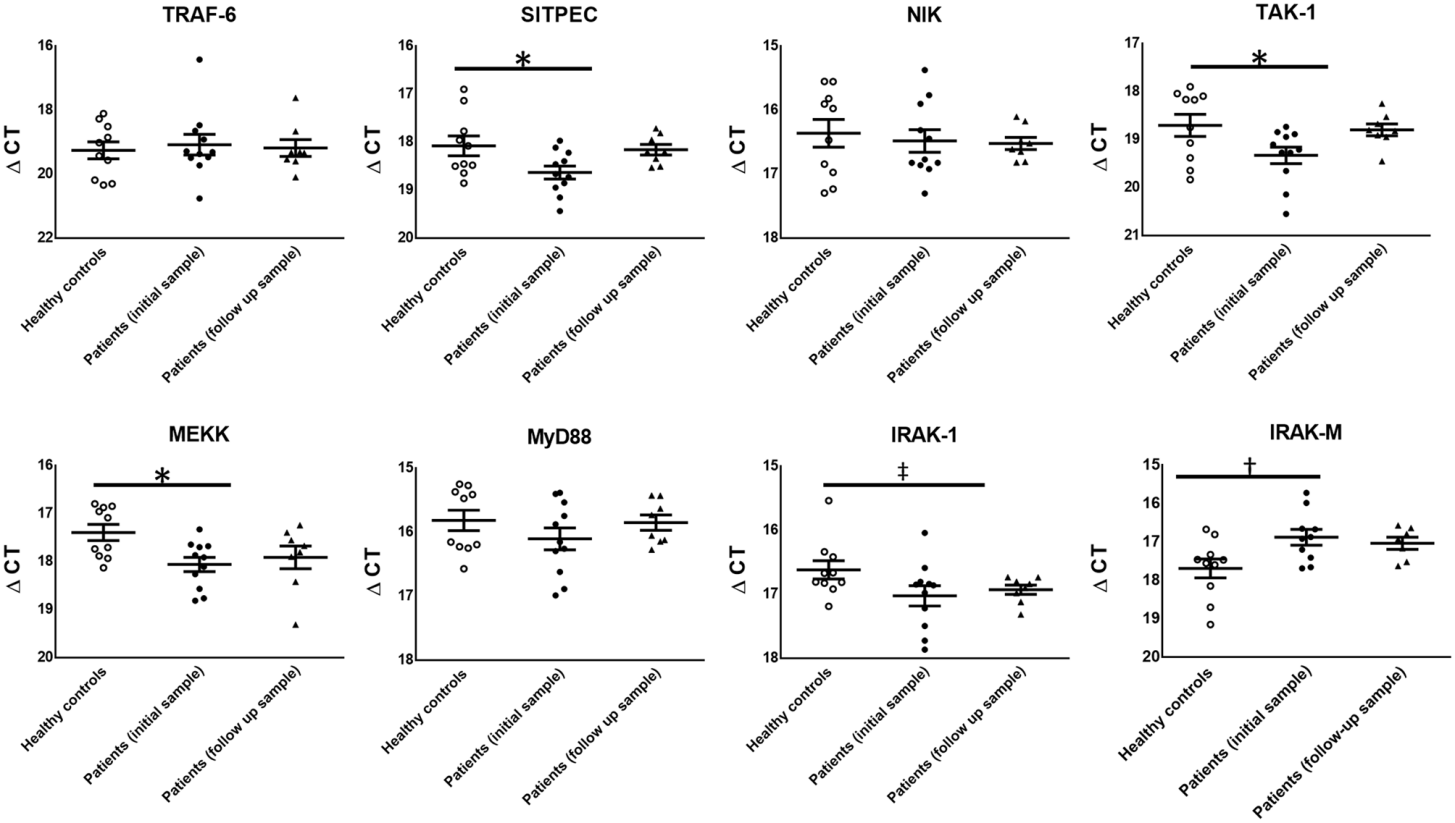
Patients with infection have decreased toll-like receptor gene expression. Peripheral blood was sampled from patients during infection and after recovery, and compared to that of healthy volunteers. CD14 monocytes were isolated from whole blood and toll-like receptor gene expression was measured with normalization to 18S. *p < 0.05, ^‡^p = 0.07, ^†^p = 0.06 ANOVA with post-hoc Holm–Sidak test.

Given the impairment of monocyte function observed in patients with infection, toll-like receptor gene expression was studied in these patients (Fig. 8). Patients with infection had decreased levels of Signaling Intermediate in Toll Pathway Evolutionarily Conserved (SITPEC), TAK1 and MEKK mRNA compared with healthy controls (p < 0.05). Levels of IRAK1, another central element to IκK activation, were lower than healthy controls (p = 0.07). At the time of infection, patients had elevated levels of monocyte IRAK-M expression, a marker of endotoxin tolerance, approaching statistical significance when compared to healthy controls (p = 0.06).

## Discussion

We adopted a model of impaired monocyte function using isolated primary human monocytes to represent the decreased cellular responsiveness seen in patients who are at very high risk. The present study builds on previous work that was used to select the time points studied^17,18^. The impaired monocytes in this model had a distinct profile based on cytokine and chemokine production with an AUC of 1.00, indicating that this impairment model profile predicted tolerant conditions in all cases. Monocytes exposed to low dose LPS had suppressed IκKα/β phosphorylation and decreased NFκB activation. Suppression of IκKα/β activation could explain the decreases in most mediators seen in the impaired monocyte, as per the loss of function experiments using IκK-16. This study also confirms that surgical patients with infection have suppressed host defences, specifically a decreased capacity for TNF-α production in response to LPS stimulation and decreased HLA-DR expression.

Many studies have demonstrated various forms of immune dysfunction following trauma, major surgery and sepsis. Among the most frequently observed aspects of host defence liabilities has been decreased monocyte HLA-DR expression^5,21,22^ and the decreased capacity for TNF-α production in response to ex-vivo LPS stimulation^6,19,23^. Marked monocyte deactivation occurs in approximately a sixth of ill patients^5^. Although the ideal biomarker for the “at risk” patient is not clear, impairments of these two facets of monocyte function have consistently predicted nosocomial infection and death in many clinical scenarios. Our findings suggest that TNF-α is the most sensitive cytokine in monocyte impairment. Despite many clinical reports, there are few that actually describe the underlying signalling mechanisms that underpin the suppressed phenotype. Recently, it was reported that patients undergoing major abdominal surgery who had increased p65 (NFκB) phosphorylation were at higher risk of exhibiting systemic inflammatory response syndrome (SIRS)^3^. Patients who develop infection appear to have alterations in the expression of NFκB subunits, with more p50-p50 homodimer formation, than the active p65-p50 heterodimer^24,25^. Van Der Poll and associates have recently reported decreased NFκB phosphorylation in response to ex-vivo monocyte LPS stimulation in patients with sepsis^7^. The findings presented herein, showing decreased IκKα/β phosphorylation in response to an LPS challenge in the impaired monocyte, are consistent with this clinical study.

A high throughput study of leukocyte gene expression in trauma patients found decreased monocyte IκKβ expression^26^. In contrast, our results show decreased IκKα mRNA and total protein levels in the impaired monocyte, but equivalent IκKβ levels. Furthermore, the level of phosphorylation of these subunits was decreased in impaired monocytes. This may be in part due to changes upstream in the pathway that prevents the function of the upstream kinase of IκKα/β. We found that levels of total and phosphorylated TAK1 were similar between naïve and impaired conditions, in contrast to Xiong et al^27^. However, in their study the levels of phosphorylated TAK1 and activation of TAK1 is diminished in tolerant THP1 monocytes, these changes were less apparent in the human monocytes. In a recent report from our group we found suppressed TRAF6 gene expression in a similar model of impairment^17^. In the current study, we undertook a pathway analysis of toll-like receptor gene expression in order to elucidate mechanisms of IκK dysregulation. This revealed a complex network of various pathways involving TAK1, NIK and MEKK1. Taken together, a TAK-1-independent mechanism resulting in decreased IκKα/β activation appears likely to involve an intermediate pathway between TRAF-6 and IκKα/β via a NIK or SITPEC/MEKK1-related mechanism. Given the observed monocyte dysfunction in patients with surgical infection, we interrogated the expression of genes implicated in this pathway in such patients. We found that the expression of SITPEC, TAK1 and MEKK1 were decreased when compared with healthy controls. IRAK-1 levels were also lower than healthy controls, although this did not reach statistical significance (p = 0.07). These findings suggest that a physiological insult can lead to dysregulation of the toll-like receptor pathway, which may underpin the molecular mechanism resulting in supressed activation of the IκK pathway leading to impaired monocyte responses.

Although the TRIF pathway is affected in other models, our model showed that although TRIF-related mediators IP-10 and IFN-β were not significantly affected by endotoxin tolerance. In fact, both mediators were upregulated in IκK inhibition, suggesting compensatory TRIF activation during IκK16 treatment. Many other studies have shown dysregulated microRNAs and other epigenetic changes that could downregulate the NFκB pathway^28^. We have previously reported that IκKα/β modulates the cytokine response through a miR-155-dependent mechanism^17^.

There is no immunoadjuvant intervention, as yet, that has consistently or reproducibly improved clinical mortality by reversing monocyte impairment. IFN-γ and GM-CSF have both been used in randomised controlled clinical trials to improve patient outcomes^29–32^. Despite effectively improving monocyte function, unchanged rates of death were seen. We believe that there are many aspects to the failure of these trials such as insufficient dosing and duration of the adjuvant as well as imperfect patient selection, given that only approximately a sixth of patients exhibit significant monocyte impairment. However, importantly there is an incomplete understanding of the underlying signalling defects involved in suppression of these important host defence parameters. Our group recently showed that while IFN-γ could restore TNF-α production and monocyte HLA-DR expression, a short exposure of IκK-16 had differing modulatory effects including decreasing TNF-α production and PD-L1 expression while preserving IL-10 levels and CD14 expression^18^. The present study indicates that suppressed IκKα/β phosphorylation is one mechanism which contributes to a blunted cytokine and chemokine response. However, given the importance of monocyte HLA-DR expression in patients and the lack of influence of IκKα/β on HLA-DR expression, there are other signalling pathways that are likely affected. We did not study the JAK-STAT1 pathway, but previous reports have shown a dysregulation of this pathway in endotoxin tolerance, which plays a role in the regulation of major histocompatibility complexes including HLA-DR^33^. A further relevant finding of our previous study is that impairment of monocyte function can lead to decreased CD14 expression, which has been confirmed in previous clinical studies^18,34^. Therefore, the present cohort of patients with infection may have further myeloid derived suppressor cells which are heterogeneous in their CD14 expression but are not fully included in the analysis due to the nature of CD14 magnetic labelling technique and would be an interesting area for further study.

Some studies investigating animal models of sepsis have focussed on IκKα/β as a therapeutic target. In subjects with haemorrhagic shock and also in caecal ligation and puncture, treatment with IκK16, a novel selective inhibitor, has led to improved end organ function^13,14^. Our study reports the use of IκK16 with human monocytes and explores the mechanism of action as well as its downstream effects on cytokine and chemokine levels. Although this adds useful data for groups investigating this line of enquiry, we believe that the inhibitory nature of this potential therapy could increase the risk of subsequent infection in the clinical setting and further translational studies should carefully consider timing and dosing of IκK16 to prevent harmful side effects.

This study has some limitations. We believe that the process of magnetic bead isolation and culture of a monocyte single cell suspension causes a minimal degree of monocyte stimulation, affecting all conditions equally. Elevated cytokine levels are noted in the unstimulated controls, and immunofluorescence images of various monocyte conditions illustrates considerable p65 co-localization within the nucleus under control conditions. However, exposure to 10 ng/mL of LPS does lead to impairment of numerous aspects of IκKα/β and NFκB signalling and is demonstrated by the distinct secretion patterns as determined by partial least squares discriminate analysis (PLS-DA).

Healthy subjects sampled are variable not just in their baseline level of monocyte function, but also by the degree to which their cells are impaired following induction of endotoxin tolerance. Our group has previously reported the differing but reproducible response to endotoxin^35^. We have, where possible, presented paired data to show within-subject differences. Although studying human subjects adds a layer of experimental variability, it preserves the clinical variability observed in patients.

We adopted a pragmatic approach to studying in-vivo impairment by accessing surgical patients with infection with variable timing of sampling. The model of endotoxin tolerance in the present study shares some of the hallmark features of impaired monocyte function (most notably decreased TNF-α in response to LPS stimulation) that are observed in the studied patients, as well as described in the literature^25,36,37^. Interestingly, we found that IκKα mRNA levels were increased in patients’ monocytes at the time of infection. These findings, however, contrast with the decreases in IκKα gene expression observed in the in-vitro model. There are various reasons that could explain this difference. It is not possible to know, at the time of first sampling, how long the patient has been unwell. There are certain variables which cannot be controlled, including the duration of the infection, nutritional status, source of infection, intervention, length of time in hospital and time until return visit for collection of subsequent sample(s). Such important factors should be considered when comparing differences in IκKα/β expression between patient samples and an in-vitro model. These factors are representative of clinical practice.

This heterogeneous set of patients had various sources of infection and were sampled at their index hospitalization and then at their subsequent follow-up in clinic. The timing of follow up sampling is influenced, in part, due to variable times of clinical recovery (Table 1). The patients were studied in the setting of complex colorectal surgical practice, and thus 5 of the 16 patients studied had inflammatory bowel disease, with 4 of these having had exposure to steroids in the preceding last 4 weeks which is a confounding factor. This is, however, representative of clinical practice and this does show relatively consistent monocyte impairment at the time of infection^15,38–40^. The partial recovery of elevated IL-10 and IRAK-M is suggestive that these markers of monocyte impairment resolve in line with clinical recovery, although this was not statistically significant. It was beyond the scope of this study to investigate whether impaired monocyte function is predictive of further infection and death in this cohort of patients, however, this correlation is well described^5,6,19,41^. These patients appeared to be in symbiosis with their infections. These clinical observations support the in-vitro model of monocyte impairment as representative of the suppressed response seen in patients. Future studies might profitably focus on determining differences in IκK phosphorylation in groups of patients with infection, including both patients who do and do not exhibit impaired monocyte function.

In conclusion, this report indicates that dysregulated IκKα expression and decreased IκKα/β phosphorylation occurs in monocyte impairment; it may be a pathological mechanism underpinning the suppressed cytokine and chemokine responses observed in response to a subsequent stimulus, similar to that seen in surgical patients with infection. Adjunctive therapy in trauma and in sepsis targeting monocyte impairment has not yet successfully improved mortality, in part due to an incomplete understanding of the affected signalling mechanisms underpinning monocyte hyporesponsiveness. Future studies should target not just restoring activation of the IκKα/β-NFκB pathway to restore cytokine and chemokine production, but also other affected pathways governing more aspects of monocyte impairment.

## Methods

### Human subjects

The study was approved by the Institutional Review Board at the University of Louisville, and all methods were performed in accordance with the appropriate guidelines and regulations. Following written informed consent in all subjects, 25 mL peripheral blood was obtained via phlebotomy (Becton Dickinson, Franklin Lakes, NJ). Two groups of human subjects were enrolled. The first group were healthy subjects (n = 16, 8 men, 8 women) not taking any immunosuppressant or anti-inflammatory medications and without any acute or chronic illnesses with ages ranging from 19 to 80 years (mean ± SD; 38.1 ± 21.8 years). The second group of individuals consisted of hospitalized surgical patients (n = 16; 9 men, 7 women, aged between 31 and 81 years, mean ± SD; 56.2 ± 15.1 years) carefully selected by one of the senior co-authors (SG) for the fact that they were clinically stable and “coexisting” with a significant infection. It was her thought that these patients could display the phenomenon of endotoxin tolerance. Phlebotomy was performed in these individuals both during the episode of infection and well after clinical recovery. The second sample was obtained 32–204 days after the initial sample (median 60 days). Table 1 provides the demographics for the patients in the study.

### Monocyte isolation

We used methods of monocyte isolation and development of endotoxin tolerance are as described in our groups previous work^17,18^. Positive magnetic selection of monocytes was performed according to the manufacturer’s protocol using Human CD14 Whole Blood Microbeads (Miltenyi Biotec, Auburn, CA). The purity of isolated monocytes was > 95% and validated using flow cytometry through the expression of CD14; cells were also stained with Trypan blue and manually counted and demonstrated > 95% viability. Monocytes were suspended in RPMI-1640 medium (Sigma Aldrich, St Louis, MO) which was supplemented with L-glutamine, 10% foetal bovine serum, and both an antimycotic and antibiotic (Thermo Fisher Scientific, Waltham, MA), incubated in a humidified atmosphere with 5% CO2 at 37 °C. All cultures were performed in 50 mL polypropylene tubes by cell suspension at a density of 1 × 10^6^ cells per condition.

In the endotoxin tolerance model, following 1 h (h) rest after isolation, cells were cultured for 16 h with media only (naïve control) or with 10 ng/mL of LPS (impaired) using *E. coli* (0111: B4; from Sigma Aldrich, St. Louis, MO). At 17 h after isolation, cells were centrifuged and re-suspended at a concentration of 0.5 × 10^6^ per mL in fresh culture media. Monocytes were then treated with an LPS challenge (100 ng/mL) to measure the inflammatory response. RNA, protein and supernatant were collected at the selected time points and stored at − 80 °C (Fig. 1A). For experiments utilising IκK-16, IκK-16 or DMSO were added to cells immediately after isolation (as described below). After 1 h following isolation, monocytes were treated with 100 ng/mL of LPS for a further 16 h prior to analysis. Monocyte viability was unaffected by naïve, LPS-treated and IκK-16 treated cellular conditions^17^.

### RNA isolation

Cells were collected at the selected time points and stored in lysis buffer at − 80 °C until later analysis. Total RNA was isolated with the MirVana™ miRNA isolation kit (Thermo Fisher Scientific, Waltham, MA). Purity and concentration of RNA were assessed by a Nanodrop N-1000 (Agilent Biosystems, Santa Clara, CA). RNA samples were utilized provided they achieved the criteria of a purity of 1.8–2.2, assessed by the absorbance ratio at 260 nm and 280 nm (A260/A280 ratio).

### Messenger RNA analysis

Gene expression was determined after complementary DNA (cDNA) was generated by reverse transcription (High-Capacity cDNA Reverse Transcription kit, Life Technologies, Foster City, CA). Specific primers for TNF-α, HLA-DR and IRAK-M quantification were used for qRT-PCR, with normalization of data to 18S as the house-keeping gene. The StepOne-Plus RealTime-PCR-System instrument was used with Taqman® Universal Master Mix (Applied Biosystems®, Foster City, CA). A normalized reporter value (ΔRn) threshold of 0.1 was used. Fold changes were determined by the ΔΔCT calculation, by comparing the impaired monocyte expression with the naïve monocyte as control^42^.

Pathway analysis was performed on previously published publically available gene expression data obtained from toll-like receptor (TLR) gene expression profiling experiments^17^. In the present study, fold changes and p-values of toll-like receptor mRNA expression comparing impaired relative to naïve conditions were uploaded to Ingenuity Pathway Analysis (IPA) database software. The pathway analyses were generated through the use of IPA (QIAGEN Inc., https://www.qiagenbioinformatics.com/products/ingenuity-pathway-analysis)^4^. The findings of the pathway analysis are expressed as color changes to demonstrate which gene targets were upregulated (red) and downregulated (green).

### Western blot analysis

Monocytes were collected and lysed by the addition of RIPA buffer containing phosphatase and protease inhibitors to prevent protein degradation (Thermo Scientific, Rockford, IL), and then stored at − 80 °C until later analysis. Samples underwent processing using a Sonifier-250 for sonification (Branson Ultrasonics, Danbury, CT) and were centrifuged for 10 min at 10,000 g. To allow equal amounts of protein in each well, samples were quantified using a bicinchoninic acid (BCA) assay (Thermo Scientific, Rockford, IL). Individual 30 µg samples were denatured in combination with 1:100 2-mercaptoethanol and 4X Bolt™ LDS sample buffer, then loaded into Bis–Tris Plus sodium dodecyl sulfate 4–12% gradient gels. Proteins were electrophoretically separated using a Bolt™ Mini Gel Tank with Bolt MES running buffer (Thermo Scientific, Waltham, MA) for 45 min at 180 mV. This included a protein size standard (Protein Western C Standard, Bio Rad, Hercules, CA). Proteins were then finally transferred over to a nitrocellulose membrane using iBlot Gel Transfer Stacks (Fisher Scientific, Hampton, NH). After transfer, membranes were then blocked in 5% non-fat dried milk in TBS-T for 1 h and underwent incubation with either rabbit or mouse anti-human primary antibodies at room temperature overnight. Primary antibody concentrations were as follows; Beta-actin 1:10,000, Vinculin 1:1,000, IκBα 1:1,000, IκKα 1:1,000, IκKβ 1:1,000, TAK-1 1:1,000, diluted in 5% non-fat dried milk with TBS-T (Cell Signaling, Danvers, MA). Phospho-IκKα/β and phospho-TAK-1 were diluted at 1:1,000 concentration in 5% Bovine Serum Albumin (BSA) in TBS-T. TBS-T was used remove unbound antibodies before membranes were then incubated with HRP-conjugated goat anti-rabbit or horse anti-mouse secondary antibodies, as appropriate, at a concentration of 1:1,000 at room temperature for 1 h. Membranes were washed further before protein bands were developed with Clarity Western ECL Substrate (Bio-Rad, Hercules, California), and captured using a ChemiDoc MP (Bio Rad, Hercules, CA). Densitometries of each protein were determined using ImageLab software (Bio Rad, Hercules, CA). For quantification, IκKα, IκKβ and phospho-IκKα/β were expressed as relative density units normalized to beta-actin as a loading control. TAK-1 activation was expressed as a ratio of phospho-TAK1 over total TAK-1, normalized to beta-actin. Vinculin was used as the loading control for IκBα quantification.

### Flow cytometry

#### Isolated monocytes from the in-vitro model

After the PBS wash, monocyte samples (100,000 cells) were stained with fluorescein isothiocyanate (FITC)-labelled anti-human CD14, phycoerythrin (PE)-labelled anti-HLA-DR antibodies (BD Biosciences, La Jolla, CA) in 100 µL of PBS at 4 °C for 25 min. Monocytes underwent centrifugation, were re-suspended and washed with Dulbecco phosphate buffered saline (Sigma Aldrich, St. Louis, MO), and fixed in 1% paraformaldehyde solution until later analysis.

Surface levels of cellular HLA-DR expression were analysed using a FACSCalibur instrument (Becton Dickinson, San Diego, CA). A total of 5,000 events were acquired at the indicated time points. Mean fluorescence intensity (MFI) for each labelled antibody was analysed on monocytes using Cell Quest (Becton Dickinson, San Diego, CA). We have previously shown that the fluorescent antibodies used do not demonstrate non-specific binding in this model^18^.

### Whole blood samples

Whole blood samples of 50 μL were stained using fluorescein isothiocyanate (FITC)-labelled anti-human CD14 and phycoerythrin (PE)-labelled anti-HLA-DR (BD Biosciences, La Jolla, CA) antibodies. After 25 min of staining at 4 °C, erythrocyte lysis was achieved with EDTA, potassium bicarbonate, and ammonium chloride (Sigma Chemical Co., St. Louis, MO) for 6 min. Cells were then washed with Dulbecco Phosphate Buffered Saline (DPBS) (Sigma Chemical Co., St. Louis, MO) and fixed in 300 μL of 1% paraformaldehyde. For analysis, samples were run using a LSR II flow cytometer (Becton Dickinson, San Diego, CA) within 48 h of venipuncture with a minimum of 20,000 events were acquired. For the gating strategy (Supplemental Fig. 1), the monocyte population was selected according to forward scatter and side scatter properties, and by gating only on CD14 positive cells to determine HLA-DR MFI using Cell Quest (Becton Dickinson, San Diego, CA).

### Cytokine analysis

Tumour necrosis factor-alpha (TNF-α), interleukin (IL)-6 and -10, and interferon-beta (IFN-β) were analysed using commercially available enzyme-linked immunosorbent cytokine assays, following the manufacturer’s instructions, in duplicate (eBioscience, San Diego, CA). For quantification, recombinant human cytokines were used to determine supernatant cytokine concentrations after appropriate dilution for accurate measurement within the standard curve. The lower limits of cytokine detection were 2 pg/mL for IL-10, 4 pg/mL for TNF-α, 8 pg/mL for IL-6, and 25 pg/mL for IFN-β.

For multiplex cytokine experiments, 25 µL supernatant sample was used per well in duplicate in pre-mixed 96 well plates using magnetic beads for 29 different cytokines and chemokines as per manufacturers recommendations (Milliplex Human Cytokine/Chemokine 29-Plex Magnetic Bead kit, EMD Millipore, Billerica, MA). Recombinant pre-mixed beads were used to create a standard curve. Quality controls 1 and 2 were used. Plates were run using a Luminex MAGPIX instrument (EMD Millipore, MA).

### IκKα/β inhibition

IκK-16 reconstituted in DMSO from stock as per manufacturer’s instructions (Sigma Aldrich, St. Louis, MO), before dilution in media to a concentration 100 mM of IκK-16 (final DMSO concentration of only 0.01%). The same DMSO concentration of 0.01% was used in control conditions. We have previously reported dose-responses up to 1,000 nM of IκK-16 for TNF-α and IL-10 production, and that cell viability is not affected by IκK-16 even at high doses^17^.

### Analysis of samples from patients with surgical infection

Whole blood monocyte surface HLA-DR expression and plasma cytokine levels were examined at baseline 0 h (unstimulated) and following ex-vivo lipopolysaccharide (LPS) stimulation (100 ng/mL) for 4 h (Fig. 7D). Plasma samples were collected after centrifugation of whole blood and stored at −80 °C until analysis. Standard individual ELISA assays were performed for TNF-α and IL-10 plasma protein concentrations and flow cytometric analysis performed both as previously described. The remaining monocytes were isolated by magnetic bead positive selection, and mRNA was extracted and analysed via qRT-PCR, to compare the ratio of normalized IκKα and IκKβ mRNA expression (using 18S) between patient and healthy control samples. The Toll-like receptor pathway genes TRAF-6, SITPEC, NIK, TAK-1, MEKK, MyD88, IRAK-1 and IRAK-M were measured by qRT-PCR using 18S as an internal control.

### Statistical analysis

Data are shown as mean ± S.E. Continuous paired data comparing two groups were analysed by Wilcoxon-signed rank test. Each donor was used as its own control. Data with multiple comparisons were analysed primarily by one-way repeated measures analysis of variance with Holm–Sidak test for post hoc analysis, or one-way analysis of variance with Holm–Sidak test for *post-hoc* analysis, where appropriate. Secondary analysis of multiplex cytokine data was undertaken by calculating the within-subject relative change, log_2_ transformation and student t-test used to calculate q-values to preserve false discovery rate^43^. A p-value of < 0.05 was considered significant. SigmaPlot was used for statistical analysis (SyStat Software, Inc., Chicago, IL) and PRISM 5.0 software (GraphPad) used for data presentation.

For partial least squares discriminatory analysis (PLS-DA), a multivariable analysis was conducted by modelling the changes in naïve and impaired conditions using R statistical software (v3.3, R Foundation for Statistical Computing). The partial least squares regression was undertaken by pairing samples by donor to allow latent component projection in a lower dimensional space. The accuracy of the model was evaluated by estimating the area under the curve (AUC)^44^.

## Supplementary information

Supplementary information.

## Data Availability

The datasets generated during and/or analysed during the current study are available from the corresponding author on reasonable request.

## References

[1] Xiao W (2011). A genomic storm in critically injured humans. J. Exp. Med..

[2] Namas RA (2016). Temporal patterns of circulating inflammation biomarker networks differentiate susceptibility to nosocomial infection following blunt trauma in humans. Ann. Surg..

[3] Lahiri R (2016). Systemic inflammatory response syndrome after major abdominal surgery predicted by early upregulation of TLR4 and TLR5. Ann. Surg..

[4] Kramer A, Green J, Pollard J, Tugendreich S (2014). Causal analysis approaches in ingenuity pathway analysis. Bioinformatics (Oxford, England).

[5] Polk HC (1986). A systematic study of host defense processes in badly injured patients. Ann. Surg..

[6] Galbraith N, Walker S, Galandiuk S, Gardner S, Polk HC (2016). The significance and challenges of monocyte impairment: for the ill patient and the surgeon. Surg. Infect..

[7] Hoogendijk AJ (2017). Sepsis patients display a reduced capacity to activate nuclear factor-kappaB in multiple cell types. Crit. Care Med..

[8] Cheadle WG (1993). The human leukocyte antigens and their relationship to infection. Am. J. Surg..

[9] Volk HD (1991). Alterations in function and phenotype of monocytes from patients with septic disease–predictive value and new therapeutic strategies. Behring Inst. Mitt..

[10] Flohe S, Scholz M (2009). HLA-DR monitoring in the intensive care unit–more than a tool for the scientist in the laboratory?. Crit. Care Med..

[11] Hinz M, Scheidereit C (2014). The IkappaB kinase complex in NF-kappaB regulation and beyond. EMBO Rep..

[12] Hacker H, Karin M (2006). Regulation and function of IKK and IKK-related kinases. Sci. STKE.

[13] Sordi R (2015). Inhibition of IkappaB kinase attenuates the organ injury and dysfunction associated with hemorrhagic shock. Mol. Med. (Camb. Mass.).

[14] Chen J (2016). IkappaB kinase inhibitor attenuates sepsis-induced cardiac dysfunction in CKD. JASN.

[15] del Fresno C (2009). Potent phagocytic activity with impaired antigen presentation identifying lipopolysaccharide-tolerant human monocytes: demonstration in isolated monocytes from cystic fibrosis patients. J. Immunol..

[16] Allantaz-Frager F (2013). Identification of biomarkers of response to IFNg during endotoxin tolerance: application to septic shock. PLoS ONE.

[17] Galbraith NJ (2017). IkappaK-16 decreases miRNA-155 expression and attenuates the human monocyte inflammatory response. PLoS ONE.

[18] Galbraith NJ (2017). The effect of IkappaK-16 on lipopolysaccharide-induced impaired monocytes. Immunobiology.

[19] Ploder M (2006). Lipopolysaccharide-induced tumor necrosis factor alpha production and not monocyte human leukocyte antigen-DR expression is correlated with survival in septic trauma patients. Shock.

[20] Munoz C (1991). Dysregulation of in vitro cytokine production by monocytes during sepsis. J. Clin. Investig..

[21] Docke WD (1997). Monocyte deactivation in septic patients: restoration by IFN-gamma treatment. Nat. Med..

[22] Galbraith NJ (2018). Temporal expression of circulating miRNA after severe injury. Surgery.

[23] Feuerecker M (2018). Early immune anergy towards recall antigens and mitogens in patients at onset of septic shock. Sci. Rep..

[24] Adib-Conquy M, Asehnoune K, Moine P, Cavaillon JM (2001). Long-term-impaired expression of nuclear factor-kappa B and I kappa B alpha in peripheral blood mononuclear cells of trauma patients. J. Leukoc. Biol..

[25] West MA, Heagy W (2002). Endotoxin tolerance: a review. Crit. Care Med..

[26] Laudanski K (2006). Cell-specific expression and pathway analyses reveal alterations in trauma-related human T cell and monocyte pathways. Proc. Natl. Acad. Sci. USA.

[27] Xiong Y (2011). Endotoxin tolerance impairs IL-1 receptor-associated kinase (IRAK) 4 and TGF-beta-activated kinase 1 activation, K63-linked polyubiquitination and assembly of IRAK1, TNF receptor-associated factor 6, and IkappaB kinase gamma and increases A20 expression. J. Biol. Chem..

[28] Biswas SK, Lopez-Collazo E (2009). Endotoxin tolerance: new mechanisms, molecules and clinical significance. Trends Immunol..

[29] Polk HC (1992). A randomized prospective clinical trial to determine the efficacy of interferon-gamma in severely injured patients. Am. J. Surg..

[30] Meisel C (2009). Granulocyte-macrophage colony-stimulating factor to reverse sepsis-associated immunosuppression: a double-blind, randomized, placebo-controlled multicenter trial. Am. J. Respir. Crit. Care Med..

[31] Bo L, Wang F, Zhu J, Li J, Deng X (2011). Granulocyte-colony stimulating factor (G-CSF) and granulocyte-macrophage colony stimulating factor (GM-CSF) for sepsis: a meta-analysis. Crit. Care.

[32] Licht AK, Schinkel C, Zedler S, Schinkel S, Faist E (2003). Effects of perioperative recombinant human IFN-gamma (rHuIFN-gamma) application in vivo on T cell response. J. Interferon Cytokine Res..

[33] Palmer CD (2016). Naturally occurring subclinical endotoxemia in humans alters adaptive and innate immune functions through reduced MAPK and increased STAT1 phosphorylation. J. Immunol..

[34] Heinzelmann M, Mercer-Jones M, Cheadle WG, Polk HC (1996). CD14 expression in injured patients correlates with outcome. Ann. Surg..

[35] Billeter AT (2012). Does clinically relevant temperature change miRNA and cytokine expression in whole blood?. J. Interferon Cytokine Res..

[36] Lopez-Collazo E, del Fresno C (2013). Pathophysiology of endotoxin tolerance: mechanisms and clinical consequences. Crit. Care.

[37] Cavaillon JM, Adib-Conquy M (2006). Bench-to-bedside review: endotoxin tolerance as a model of leukocyte reprogramming in sepsis. Crit. Care.

[38] Turrel-Davin F (2011). mRNA-based approach to monitor recombinant gamma-interferon restoration of LPS-induced endotoxin tolerance. Crit. Care.

[39] van ’t Veer C (2007). Induction of IRAK-M is associated with lipopolysaccharide tolerance in a human endotoxemia model. J. Immunol..

[40] Escoll P (2003). Rapid up-regulation of IRAK-M expression following a second endotoxin challenge in human monocytes and in monocytes isolated from septic patients. Biochem. Biophys. Res. Commun..

[41] Sauaia A, Moore FA, Moore EE (2017). Postinjury inflammation and organ dysfunction. Crit. Care Clin..

[42] Livak KJ, Schmittgen TD (2001). Analysis of relative gene expression data using real-time quantitative PCR and the 2(-Delta Delta C(T)) Method. Methods (San Diego Calif.).

[43] Hochberg Y, Benjamini Y (1990). More powerful procedures for multiple significance testing. Stat. Med..

[44] Hastie T, Tibshirani R, Friedman JH (2009). The Elements of Statistical Learning: Data Mining, Inference, and Prediction.

