# Supplementary material for: The role and function of IκKα/β in monocyte impairment
Source: Sci Rep. 2020 Jul 22;10:12222. doi: 10.1038/s41598-020-68018-x (PMC7376008; doi:10.1038/s41598-020-68018-x)
Supplement: Supplementary file 1 — Supplementary information. [file 41598_2020_68018_MOESM1_ESM.docx]

**Supplementary Material**

The role and function of IκKα/β in monocyte impairment

Norman J. Galbraith^1^, Sarah A. Gardner^1^, Samuel P. Walker^1^, Patrick Trainor ^2^, Jane V. Carter^1^, Campbell Bishop^1^, Harshini Sarojini^1^, Stephen O’Brien^1^, Aruni Bhatnagar^2^, Hiram C. Polk, Jr.^1 *^, Susan Galandiuk^1^.

^1^ From the Price Institute of Surgical Research, Department of Surgery, University of Louisville School of Medicine, Louisville, KY.

^2^ Diabetes and Obesity Center, Institute of Molecular Cardiology, University of Louisville School of Medicine, Louisville, KY.

* Corresponding Author. Address correspondence and reprint requests to Dr. Hiram C. Polk, Jr., Department of Surgery University of Louisville School of Medicine, Louisville, KY 40292, USA. Email: hcpolk01@louisville.edu; Phone: (502) 852-1897; Fax (502) 852-8915.

**Supplemental Table 1.** Effect of monocyte impairment and IκK inhibition on cytokine and chemokine production. Mean ± SEM shown. P-values reflect Repeated measures ANOVA with post hoc Holms-Sidak test. Analytes out with upper and lower detection limits are excluded. IL-6 and IFN-β data obtained by individual ELISA rather than Multiplex assay. *p = 0.08.

**Supplemental Figure 1.** Gating strategy for flow cytometric analysis of patient samples.

**Supplementary Figure 2.** Signaling networks influenced by monocyte impairment based on cytokine/chemokine dysregulation

**Supplementary Figure 3.** Inhibition of IκK did not influence IRAK-M expression.

**Supplementary materials** – Uncropped Western Blots

| **Cytokine** | **Unstimulated** | **Naïve** | **Impaired** | **IκK-16** | **p**  **Impaired vs. Naïve** | **p**  **IκK-16 vs. Naive** |
| --- | --- | --- | --- | --- | --- | --- |
| ***Pro-inflammatory cytokines*** | | | | | | |
| **TNF-α** | 405.5 (86.6) | 1325.8 (300.6) | 671.3 (140.4) | 509.8 (115.6) | <0.05 | <0.05 |
| **IL-1α** | 1205.4 (280.2) | 1419.3 (360.7) | 2054.8 (522.9) | 182.4 (42.5) | <0.05* | <0.05 |
| **IL-1β** | 683.1 (134.8) | 1245.9 (230.0) | 1050.5 (253.1) | 56.08 (13.8) | 0.08 | <0.05 |
| **IL-12p70** | 12.2 (1.3) | 15.8 (1.7) | 14.0 (1.7) | 8.63 (1.0) | <0.05 | <0.05 |
| **IL-6** | 11310.1 (2949.1) | 16899.2 (3288.1) | 14384.4 (3066.9) | 764.6 (314.9) | <0.05 | <0.05 |
| ***Anti-inflammatory cytokines*** | | | | | | |
| **IL-10** | 4446.3 (1157.6) | 6692.7 (1398.9) | 4785.9 (1032.5) | 1279.5 (272.1) | <0.05 | <0.05 |
| **IL-1RA** | 200.8 (32.3) | 252.6 (41.3) | 215.1 (32.1) | 218.3 (55.7) | NS | NS |
| ***Chemokines*** | | | | | | |
| **MCP-1** | 7647.6 (1238.3) | 6770.5 (1219.9) | 5287.8 (1156.7) | 5482.5 (1153.2) | <0.05 | NS |
| **MIP-1β** | 4699.8 (834.2) | 7318.2 (1104.8) | 5534.1 (835.6) | 3524.4 (755.7) | <0.05 | <0.05 |
| **IP-10** | 116.7 (30.2) | 105.2 (31.9) | 60.2 (12.9) | 1582.2 (455.4) | NS | <0.05 |
| **Eotaxin** | 25.3 (1.4) | 27.3 (1.7) | 27.8 (2.1) | 21.2 (1.6) | NS | <0.05 |
| **IL-8** | 14150.2 (462.5) | 14283.3 (597.9) | 14000.0 (833.5) | 11606.2 (1097.0) | NS | <0.05 |
| ***Growth factors*** | | | | | | |
| **G-CSF** | 3987.7 (997.1) | 5121.1 (1234.5) | 5199.3 (1160.4) | 162.9 (37.8) | NS | <0.05 |
| **GM-CSF** | 246.3 (78.8) | 384.0 (86.2) | 305.9 (69.2) | 29.5 (7.5) | <0.05 | <0.05 |
| **EGF** | 20.9 (1.3) | 22.8 (1.7) | 23.0 (1.4) | 18.3 (1.4) | NS | <0.05 |
| **VEGF** | 148.3 (10.8) | 175.0 (13.1) | 160.1 (12.0) | 121.1 (13.2) | <0.05 | <0.05 |
| ***Other immunoregulatory cytokines*** | | | | | | |
| **IFN-α2** | 52.4 (4.9) | 68.8 (6.0) | 59.0 (5.1) | 39.2 (4.1) | <0.05 | <0.05 |
| **IFN-β** | 6.4 (1.8) | 7.0 (1.9) | 7.2 (1.7) | 10.9 (2.5) | NS | 0.051 |
| **IFN-γ** | 34.9 (4.0) | 38.8 (3.9) | 38.0 (3.6) | 19.5 (2.7) | NS | <0.05 |
| **TNF-β** | 6.8 (0.7) | 8.2 (0.8) | 7.1 (0.6) | 5.8 (0.6) | <0.05 | <0.05 |
| **IL-4** | 47.5 (4.5) | 53.7 (4.8) | 53.7 (5.0) | 37.0 (3.5) | NS | <0.05 |
| **IL-7** | 26.5 (2.3) | 30.7 (2.6) | 28.2 (2.6) | 19.0 (2.2) | <0.05 | <0.05 |

**Supplemental Table 1.** Effect of monocyte impairment and IκK inhibition on cytokine and chemokine production. Mean ± SEM shown. P-values reflect Repeated measures ANOVA with post hoc Holms-Sidak test. Analytes out with upper and lower detection limits are excluded. IL-6 and IFN-β data ran by ind ividual ELISA rather than Milliplex assay.


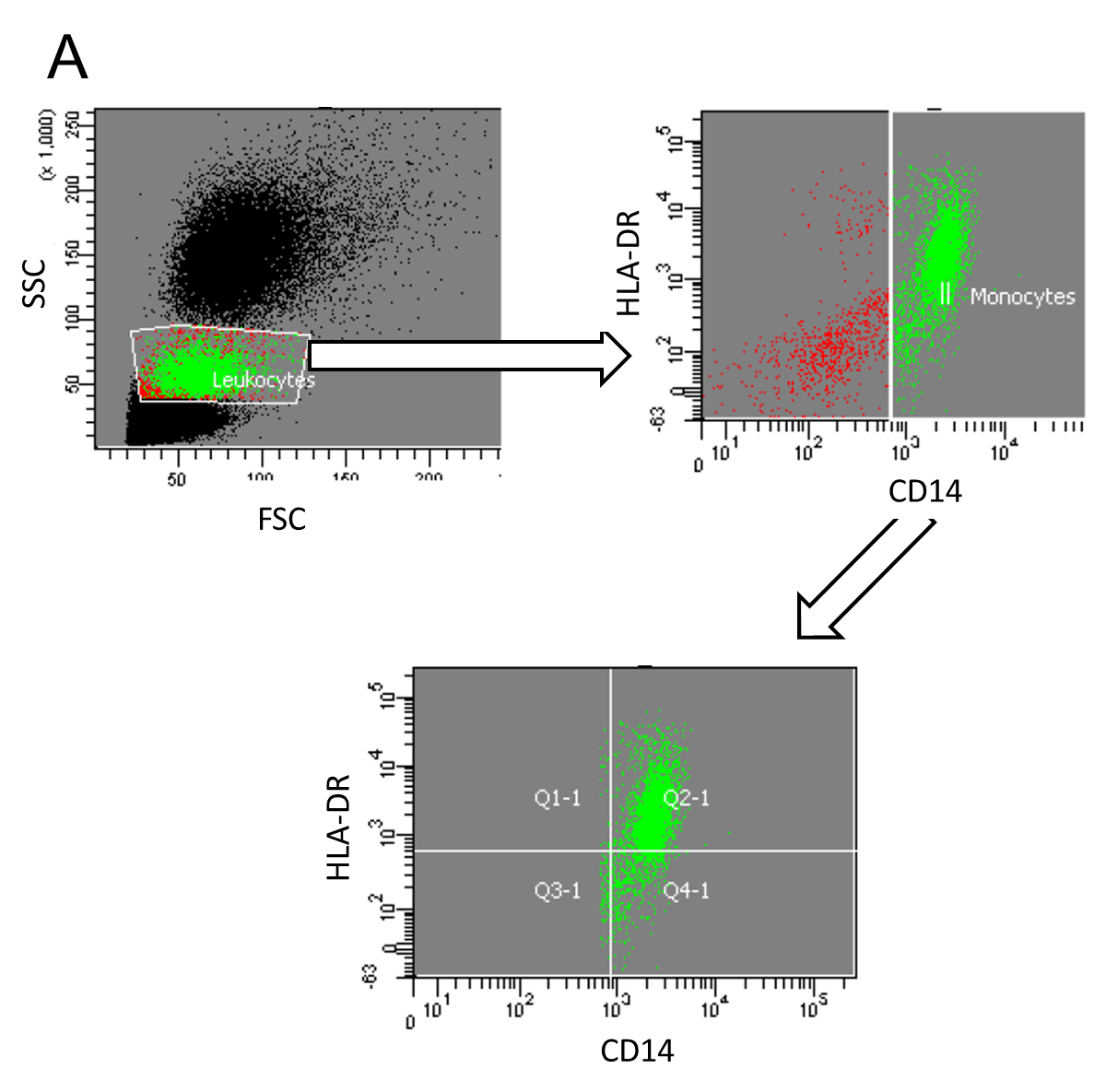


**Supplemental Figure 1.** Gating strategy for flow cytometric analysis of patient samples. *FSC, forward scatter, SSC, side scatter.*


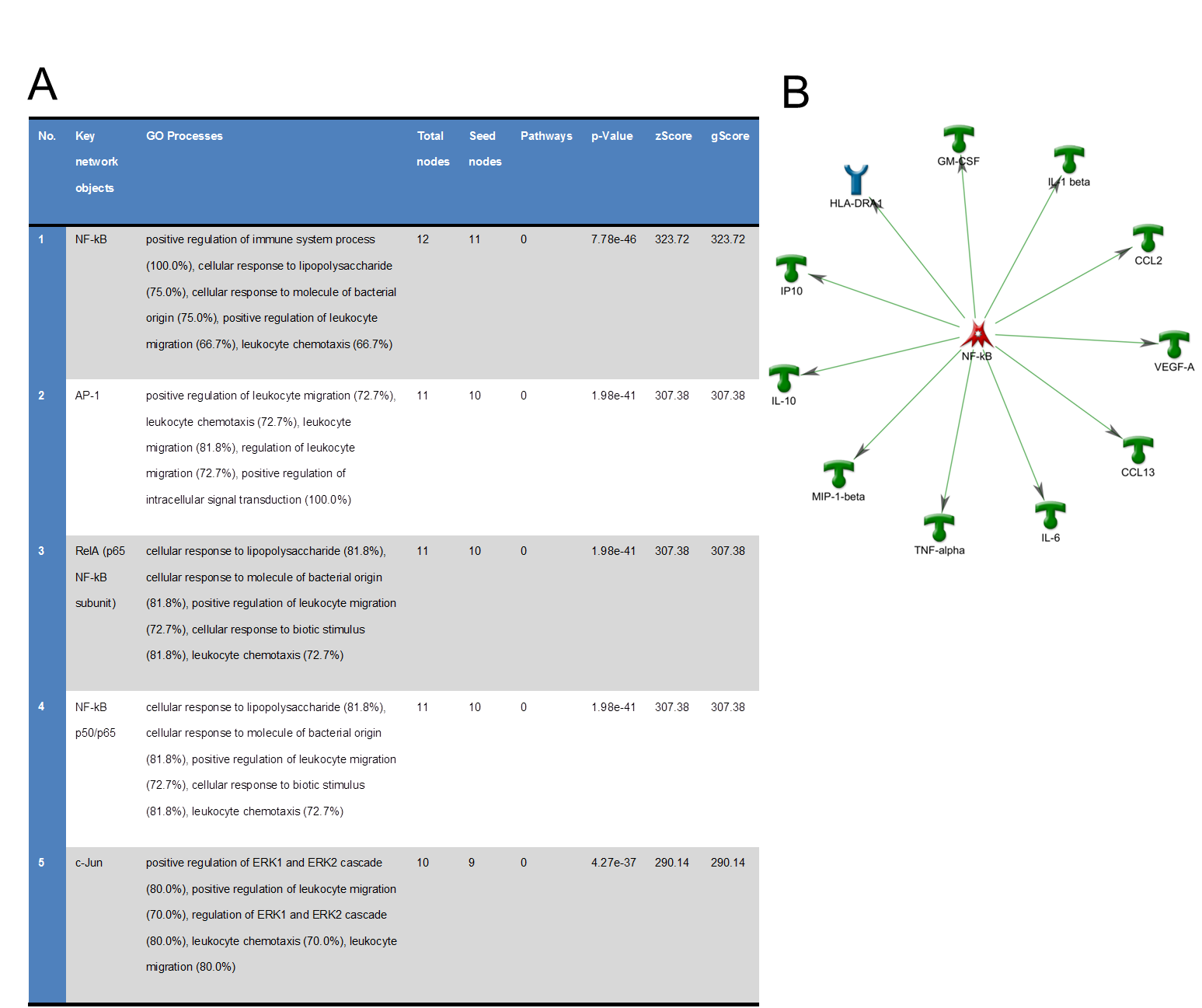


**Supplementary Figure 2.** Signaling networks influenced by monocyte impairment based on cytokine/chemokine dysregulation. Certain data included herein are derived from MetaCore (Feb 2020) data, provided by Clarivate. MetaCore and CLARIVATE are trademarks of their respective owners and used herein with permission.

**Supplementary Figure 3.** Inhibition of IκK did not influence IRAK-M expression. Primary monocytes were cultured for 16 h in conditions of LPS 100 ng/mL and DMSO, or LPS 100 ng/mL and 100 nM IκK-16. IRAK-M mRNA was determined using qRT-PCR with 18S as an internal control.

**Supplementary Materials – Uncropped Western Blots**

**Figure 3A**


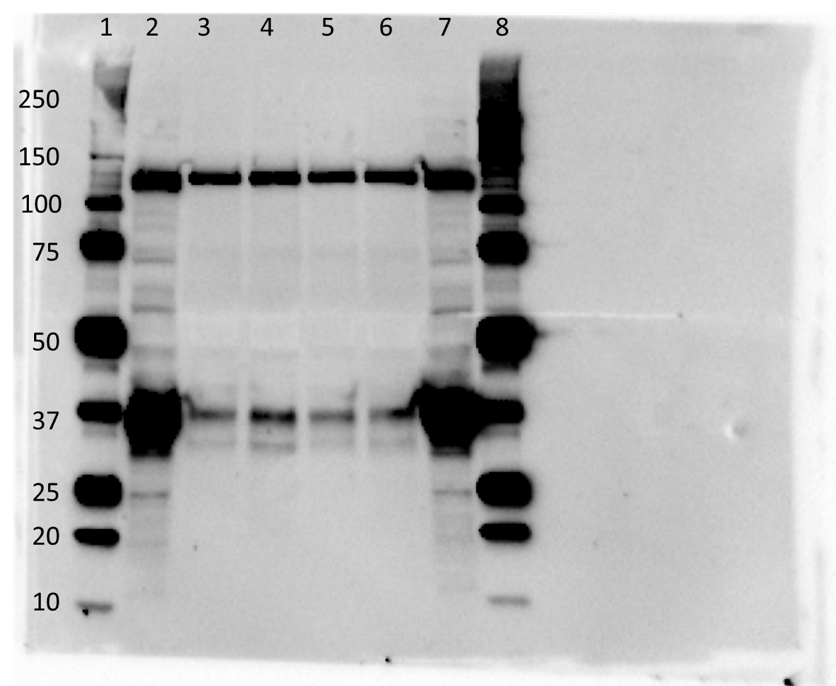


Uncropped Western Blot for IκBα. Lanes 1 & 8 show size standards (kDa). Lanes 2 & 7 show positive control for vinculin (130 kDa) and IκBα (39 kDa). Lanes 3 and 4 show naïve and impaired conditions, respectively, for Donor A. Lanes 5 and 6 show naïve and impaired conditions, respectively, for Donor B.

**Figure 3B**


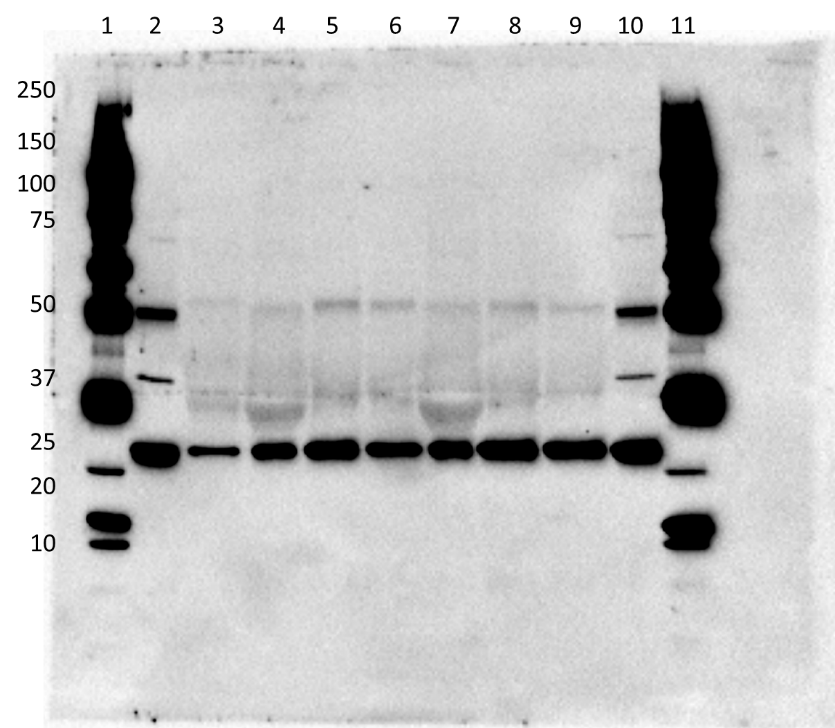


Uncropped Western Blot for IκK-α. Lanes 1 & 12 show size standards. Lanes 2 & 11 show positive controls for beta actin (42 kDa) and IκK-α (87 kDa). Lanes 5 and 6 show naïve and impaired conditions for a given donor.

**Figure 3C**


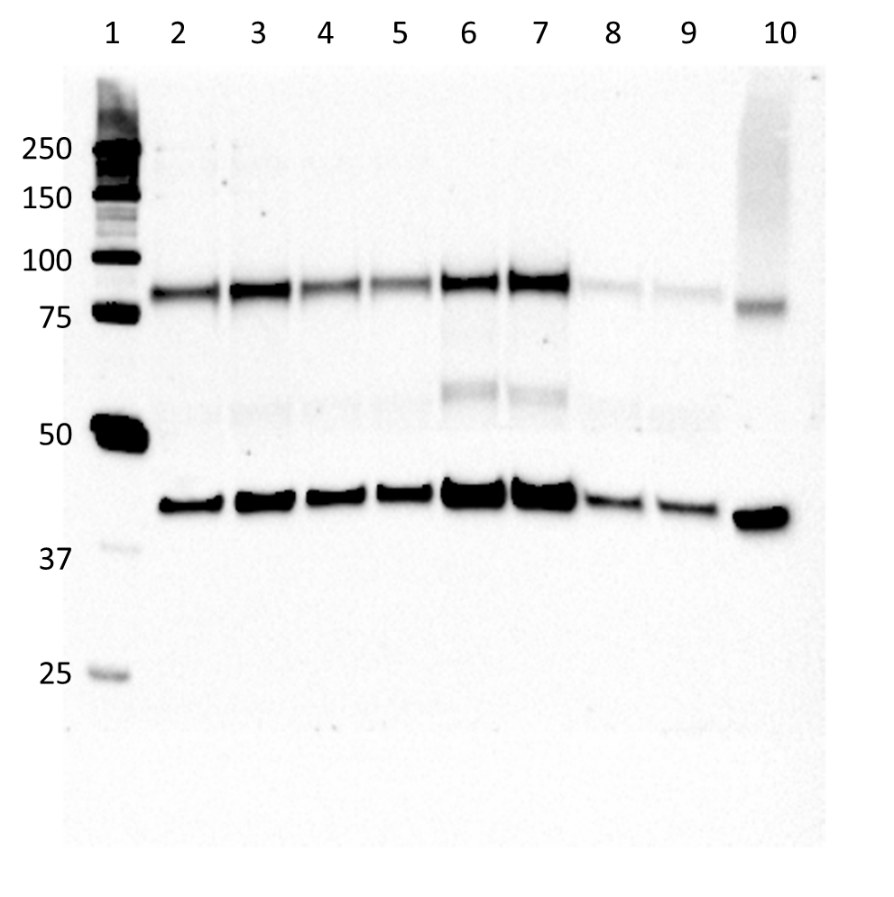
 Uncropped Western Blot for IκK-β. Lane 1 shows size standards. Example donor samples for naïve and impaired conditions are shown in lanes 2 & 3, 4 & 5, 6 & 7 and 8 & 9. Lane 10 shows positive control for beta actin (42 kDa) and IκK-β (87 kDa).

**Figure 3F**

**
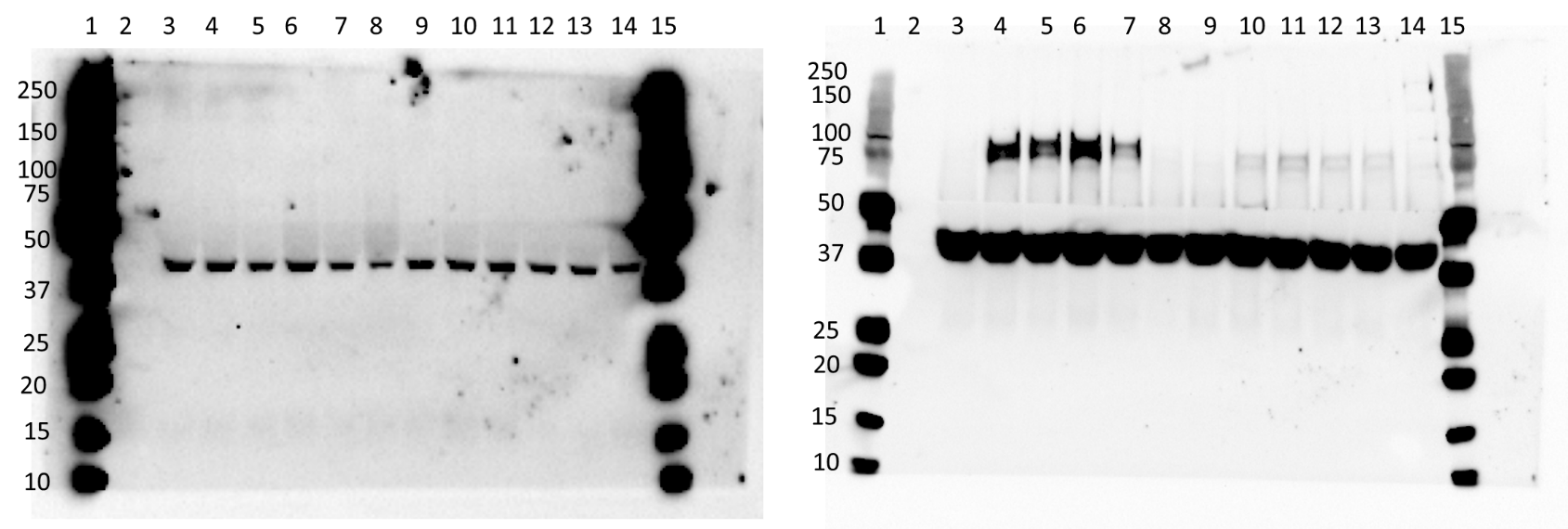
**

Uncropped Western Blot images for phosphorylated IκKα/β. Each gel shows naïve conditions (lanes 3 – 8) and impaired conditions (lanes 9 – 14) for an example donor. Size standards are shown in late 1 and 15 on each gel. Respective lanes for naïve and impaired gels are as follows for each time point after 100 ng/mL LPS stimulation; 0 mins (Lane 3 / 9), 15 min (Lane 4 / 10), 30 min (Lane 5 / 11), 45 min (Lane 6 / 12), 60 min (7 / 13), 120 min (8 / 14). Each gel shows the same membrane at different exposure times.

**
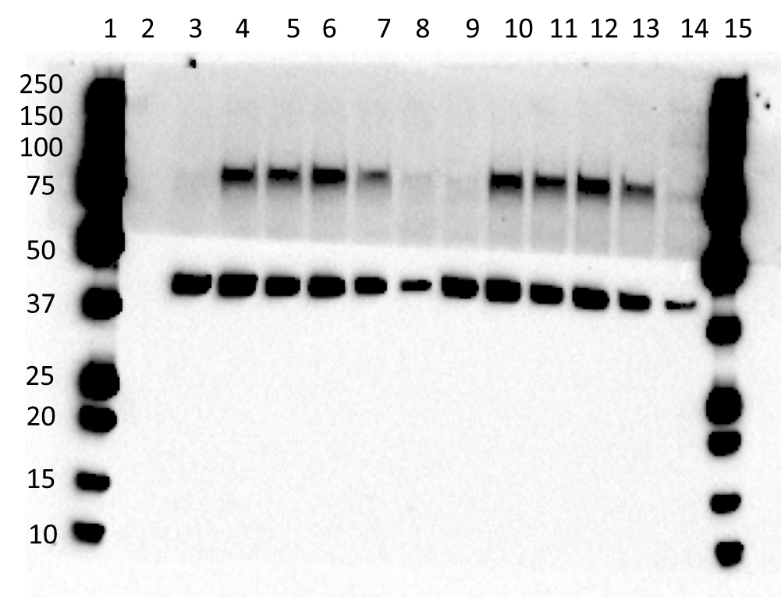
Figure 4A**

**
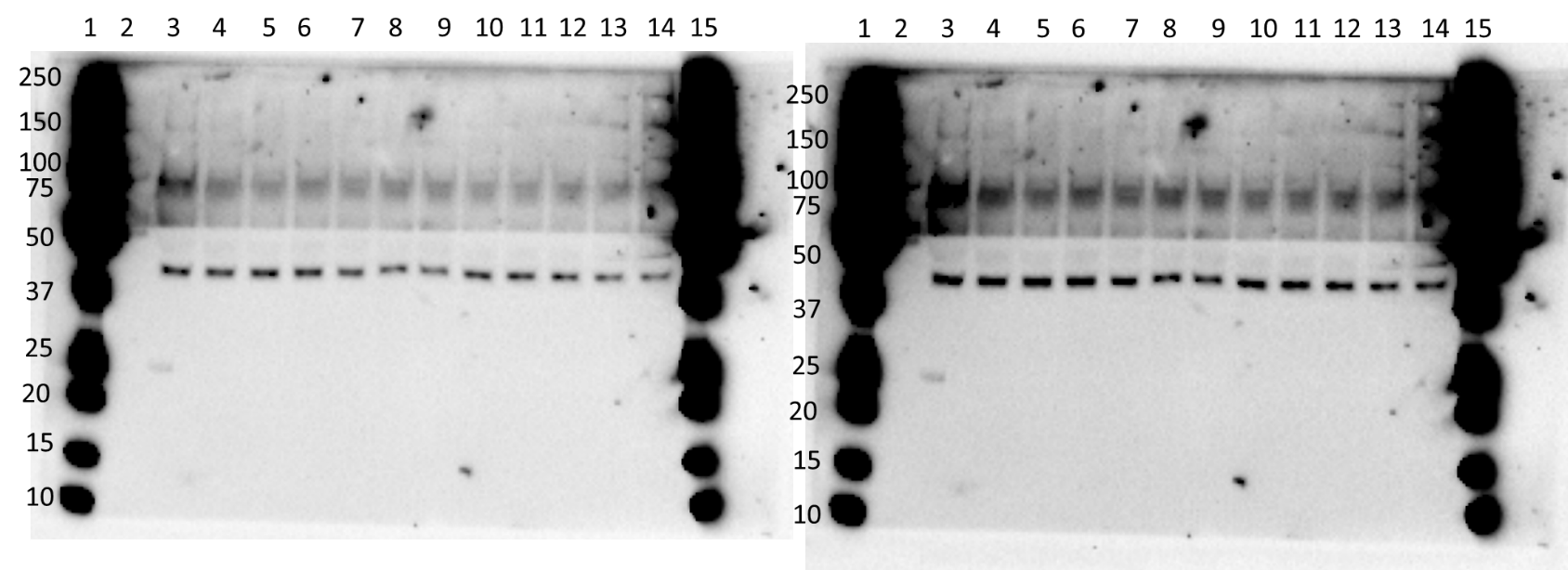
**

Uncropped Western Blot for phosphorylated and total TAK-1 expression. The gel on the left is for phosphorylated TAK-1, and the gel on the right are for total TAK-1. Size standards are shown in late 1 and 15 on each gel. Respective lanes for naïve and impaired gels are as follows for each time point after 100 ng/mL LPS stimulation; 0 mins (Lane 3 / 9), 15 min (Lane 4 / 10), 30 min (Lane 5 / 11), 45 min (Lane 6 / 12), 60 min (7 / 13), 120 min (8 / 14). Protein bands are seen for beta actin (42 kDa) and for phosphorylated and total TAK-1 for left and right gels (both 82 kDa), respectively.

**Figure 5A**

**
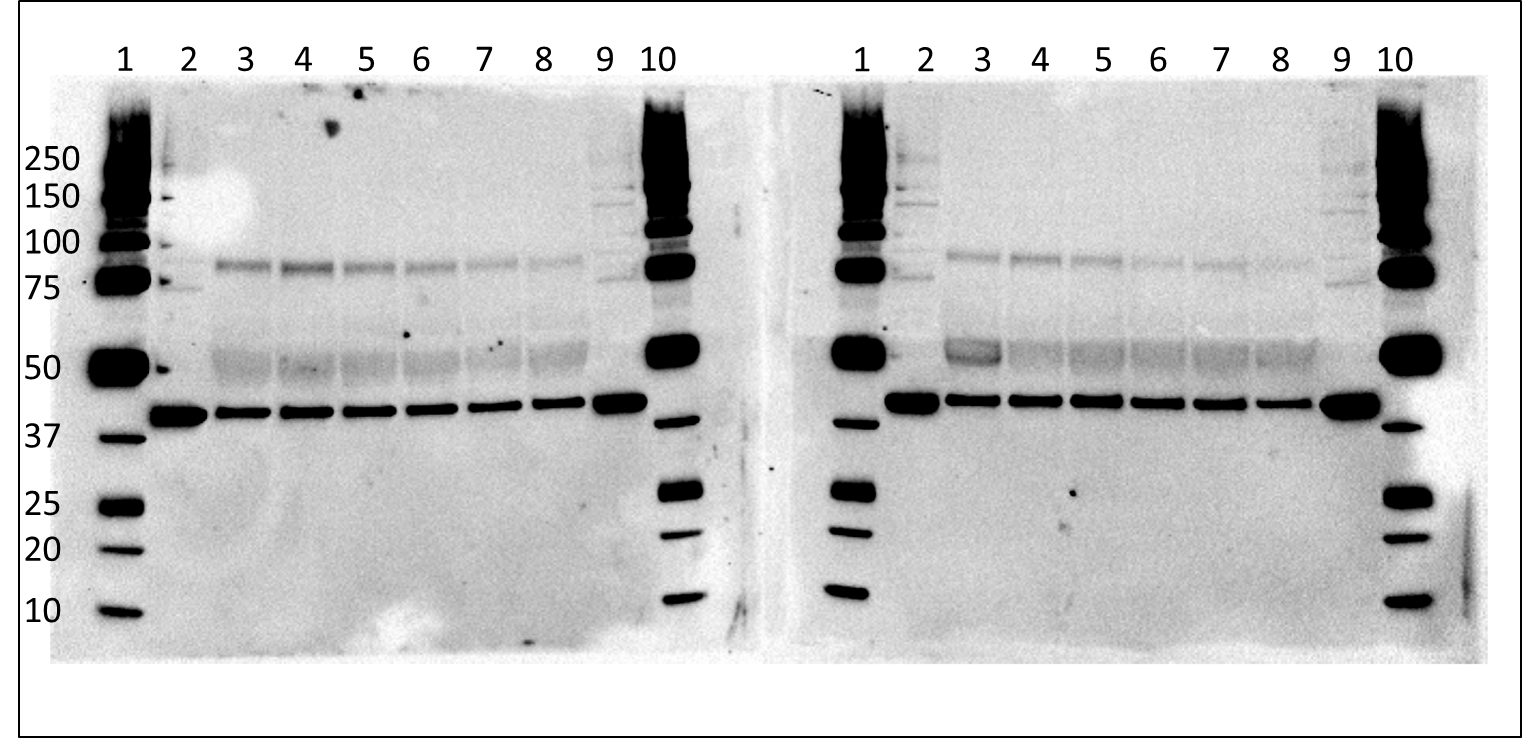
**

Uncropped Western Blot for the effect of IκK-16 on IκKα/β expression. The gel on the left demonstrates a time course for untreated monocytes (LPS stimulation only) compared with the gel on the right demonstrating IκK-16 treatment and LPS stimulation. Lanes 1 and 10 show size standards. Time points in relation to LPS stimulation are shown as follows; 0 min (lane 3), 15 min (lane 4), 30 min (lane 5), 45 min (lane 6), 60 min (lane 7), and 120 min (lane 8).
